# Enantioselective total synthesis of (−)-myrifabral A and B†

**DOI:** 10.1039/d0sc01141j

**Published:** 2020-04-21

**Authors:** Tyler J. Fulton, Anthony Y. Chen, Michael D. Bartberger, Brian M. Stoltz

**Affiliations:** Warren and Katharine Schlinger Laboratory for Chemistry and Chemical Engineering, Division of Chemistry and Chemical Engineering, California Institute of Technology Pasadena CA 91125 USA stoltz@caltech.edu; 1200 Pharma LLC 844 East Green Street, Suite 204 Pasadena CA 91101 USA michael.bartberger@1200pharma.com

## Abstract

A catalytic enantioselective approach to the *Myrioneuron* alkaloids (−)-myrifabral A and (−)-myrifabral B is described. The synthesis was enabled by a palladium-catalyzed enantioselective allylic alkylation, that generates the C(10) all-carbon quaternary center. A key *N*-acyl iminium ion cyclization forged the cyclohexane fused tricyclic core, while vinyl boronate cross metathesis and oxidation afforded the lactol ring of (−)-myrifabral A. Adaptation of previously reported conditions allowed for the conversion of (−)-myrifabral A to (−)-myrifabral B.

## Abstract 2

A catalytic enantioselective approach to the *Myrioneuron* alkaloids (−)-myrifabral A and (−)-myrifabral B is described.

## Graphical abstract

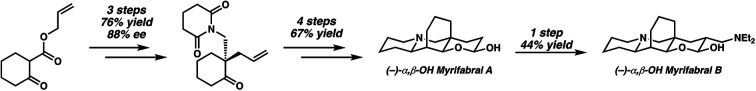

## Introduction

**Fig. 1:**
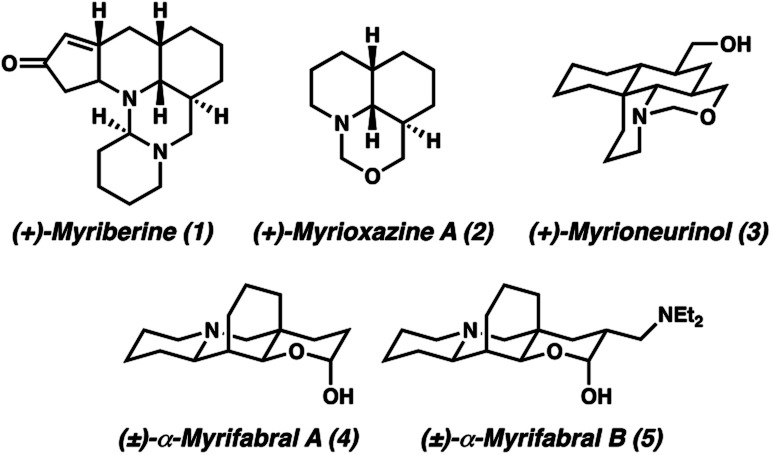
Representative *Myrioneuron* alkaloids.

The *Myrioneuron* alkaloids are a small, yet growing family of structurally diverse polycyclic (tri-, tetra-, penta-, hexa-, and decacyclic) alkaloids believed to share a common biosynthetic origin from lysine (Fig. 1).^1^ The first *Myrioneuron* alkaloids from *Myrioneuron nutans* were reported in 2002, with altogether 10 structures reported to date.^2^ Since 2013, many new alkaloids have been isolated from *Myrioneuron faberi*,^3^*Myrioneuron tonkinesis*,^4^ and *Myrioneuron effusum*.^5^ In addition to their interesting structural features, a number of these alkaloids possess a range of biological activities such as antimalarial properties, KB cell cytotoxicity, antimicrobial, and hepatitis C virus (HCV) replication inhibition.^1–5^ Despite possessing promising biological properties and synthetically attractive motifs, relatively few of these alkaloids have been prepared by total synthesis efforts.^2*d*,*e*,*f*,6^

We became interested in (±)-myrifabral A (**4**) and (±)-myrifabral B (**5**) in particular due to their unique cyclohexane fused octahydroquinolizine skeletons which contain four contiguous stereogenic centers, including an all-carbon quaternary center embedded in the cyclohexane fusion. Interestingly, both **4** and **5** are isolated as racemic mixtures of α- and β-hydroxy epimers. Even as racemates, these clusters display promising HCV replication inhibition (EC_50_ = 4.7 μM for (±)-α,β-OH-**4** and 2.2 μM (±)-α,β-OH-**5**, respectively) with significantly reduced liver cell cytotoxicity compared to commercial pharmaceutical HCV drug telaprevir (EC_50_ = 0.09 μM).^3*c*^ She *et al.* recently reported a rapid total synthesis of (±)-α,β-OH-**4** and (±)-α,β-OH-**5**, however, no asymmetric approaches have been disclosed to date.^6*c*^ To enable further studies of these alkaloids in each enantiomeric series, we report herein a short, catalytic enantioselective synthesis of (−)-α,β-OH-**4** and (−)-α,β-OH-**5**.

**Scheme 1:**
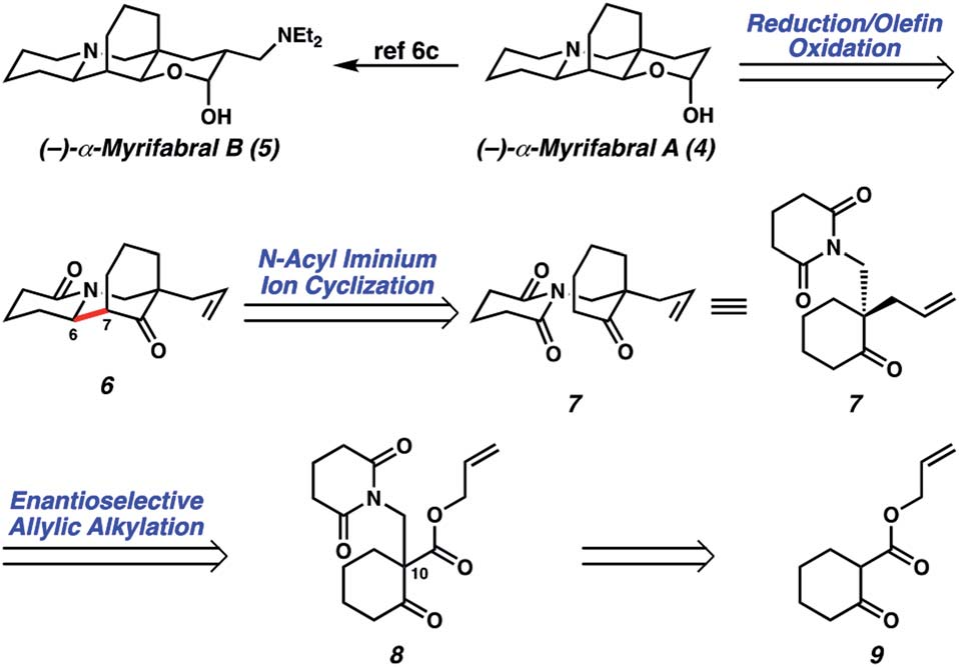
Retrosynthetic analysis of (−)-myrifabral A.

In devising our strategy, we targeted (−)-myrifabral A (**4**), which can be directly converted to (−)-myrifabral B (**5**), as reported by She (Scheme 1).^6*c*^ Retrosynthetically, we envisioned (−)-myrifabral A (**4**) could be simplified to tricyclic lactam (**6**). Importantly, the versatile ketone, allyl, and lactam functional handles in tricyclic lactam **6** provide ample opportunity for future diversification of the natural product scaffold for medicinal chemistry efforts and potential derivative synthesis. We envisioned the critical C(6)–C(7) bond could arise by means of a diastereoselective *N*-acyl iminium ion cyclization of enantioenriched ketone **7**.^7^ Finally, the C(10) all-carbon quaternary center could be forged in an enantioselective manner by means of asymmetric allylic alkylation of glutarimide **8**. In turn, glutarimide **8** could be prepared from β-ketoester **9**.

## Results and discussion

**Scheme 2:**
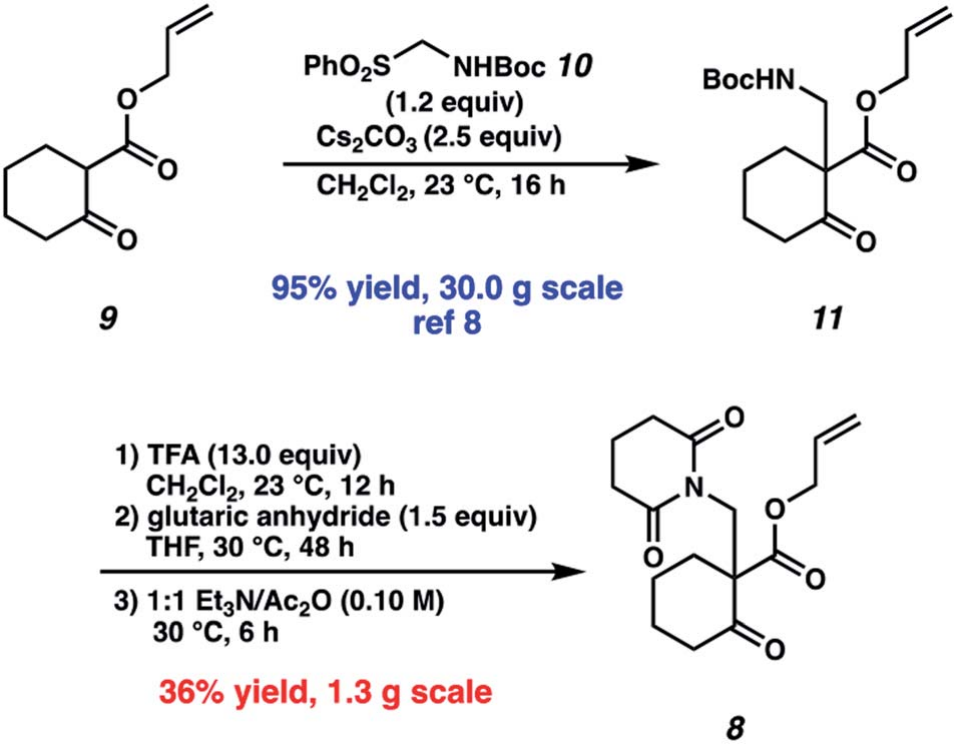
Initial synthesis of glutarimide **8**.

Our synthetic efforts commenced with the preparation of glutarimide **8** (Scheme 2). Alkylation of β-ketoester **9** (ref. 8) with sulfonylmethyl carbamate **10** in the presence of Cs_2_CO_3_ proceeded smoothly on a 30.0 g scale to afford β-aminoketone **11** in 95% yield.^9^ Elaboration of the Boc-protected amine to glutarimide **8** with standard protocols^10^ was low yielding and required three separate reactions. This inspired us to develop a more practical, one-pot procedure to enable the installation of the glutarimide moiety (Table 1). Initially, trifluoroacetic acid mediated conditions were explored, with the most promising results observed in 3 : 1 DMF/TFA at refluxing temperature (entry 1) and 3 : 1 1,4-dioxane/TFA (entry 2). Further investigation revealed that stoichiometric boric acid could mediate the transformation in excellent yield in xylenes at 140 °C (entry 3). While aryl boronic acids^11^ and boric acid^12^ have been demonstrated as effective catalysts for amidation of carboxylic acids with amines, we were surprised to observe concomitant Boc removal, amidation, and glutarimide cyclization. Control experiments revealed the reaction only proceeds with the complete set of reagents (entries 4–6). Furthermore, Boc protected β-aminoketone **11** was stable to xylenes at reflux for 120 h (entry 7). Additional examination revealed catalytic boric acid (10 mol%) with 2 equivalents of glutaric anhydride (**12**) performed equally well as our best conditions (entry 8). Replacing glutaric anhydride (**12**) with glutaric acid (**13**) as a cheaper alternative did not affect the reaction time or yield (entry 9). Pleasingly, the reaction time could be reduced by utilizing catalytic PhB(OH)_2_ as a soluble boronic acid (entry 10), although this benefit was tempered on larger scales (entry 11). Optimal results were obtained by utilizing the electron deficient catalyst 4-CF_3_PhB(OH)_2_ (entry 12).

**Table 1:** Development of a one-pot conversion of Mannich adduct **11** to glutarimide **8**

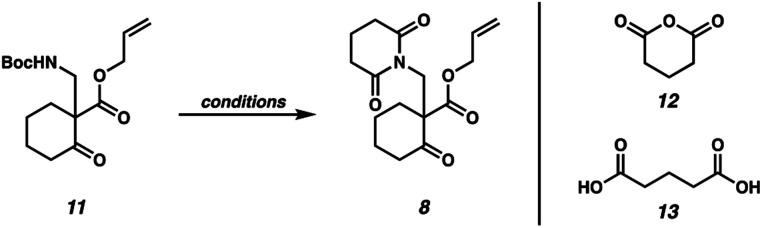		
Entry	Conditions^a^	% yield^b^
1	3 : 1 DMF/TFA (0.10 M), **12** (4 equiv.), reflux, 48 h	60

2	3 : 1 1,4-dioxane/TFA (0.10 M), **12** (4 equiv.), reflux, 48 h	70

3	B(OH)_3_ (3.0 equiv.), **12** (4 equiv.), xylenes, Dean–Stark, reflux, 36 h	90–95

4	B(OH)_3_ (3.0 equiv.) xylenes, Dean–Stark, reflux, 72 h	>95% **11**

5	**12** (4 equiv.), xylenes, Dean–Stark, reflux, 72 h	>95% **11**

6	**13** (4 equiv.), xylenes, Dean–Stark, reflux, 72 h	>95% **11**

7	Xylenes, Dean–Stark, reflux, 120 h	>95% **11**

8	B(OH)_3_ (10 mol%), **12** (2 equiv.), xylenes, Dean–Stark, reflux, 36 h	90–95

9	B(OH)_3_ (10 mol%), **13** (2 equiv.), xylenes, Dean–Stark, reflux, 36 h	90–95

10	PhB(OH)_2_ (10 mol%), **13** (2 equiv.), xylenes, Dean–Stark, reflux, 24 h	90–95

11	PhB(OH)_2_ (10 mol%), **13** (2 equiv.), xylenes, Dean–Stark, reflux, 36 h^c^	90–95

12	4-CF_3_PhB(OH)_2_ (10 mol%), **13** (2 equiv.), xylenes, Dean–Stark, reflux, 24 h^c^	90–95

a Reaction performed on a 0.16 mmol scale unless otherwise stated.

b Isolated yield; ranges reflect yields obtained from 3–4 reactions.

c Reaction performed on a 0.80 mmol scale.

**Scheme 3:**
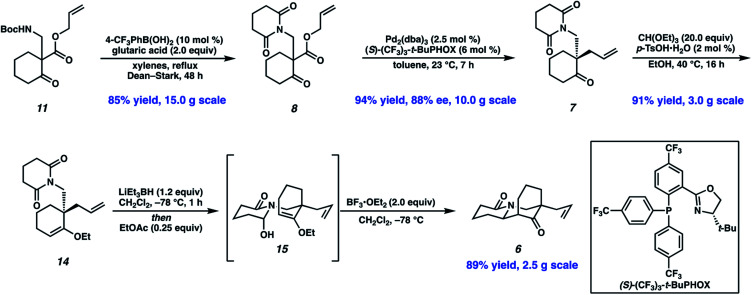
Enantioselective synthesis of tricyclic lactam **6**.

These optimized conditions for elaboration of Boc protected β-aminoketone **11** to glutarimide **8** performed well on a 15.0 g scale, enabling us to press forward in our synthetic campaign (Scheme 3). Palladium-catalyzed decarboxylative asymmetric allylic alkylation of glutarimide **8** established the C(10) all-carbon quaternary center, affording ketone **7** in 94% yield and 88% ee. The absolute configuration of the all-carbon quaternary center was established as (*S*) *via* experimental and computational vibrational circular dichroism (VCD) and optical rotation analyses.^13^ To affect the key *N*-acyl iminium ion cyclization, the ketone was first protected as ethyl vinyl ether **14**. A one-pot protocol was then employed to affect mono-reduction of the glutarimide with LiEt_3_BH followed by BF_3_·OEt_2_ mediated *N*-acyl iminium ion cyclization of intermediate *N*-acyl hemiaminal **15**, furnishing tricyclic lactam **6** as a single diastereomer in 89% yield.

**Scheme 4:**
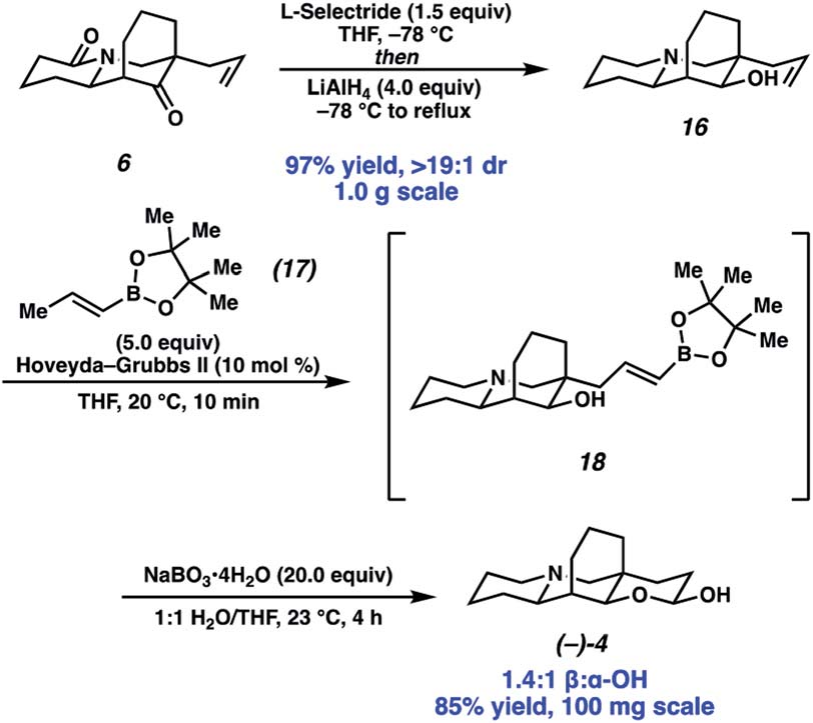
End game for (−)-myrifabral A.

**Scheme 5:**
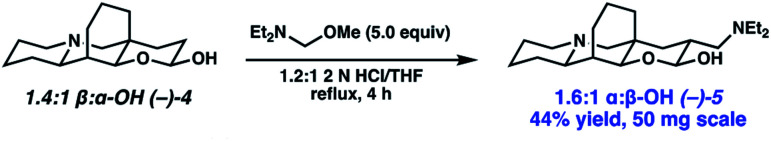
Synthesis of (−)-myrifabral B.

Completion of the synthesis required reduction of the lactam and ketone and elaboration of the terminal olefin to an aldehyde (Scheme 4). Toward that end, a one-pot procedure was developed wherein the ketone was first reduced by l-selectride with exceptional diastereoselection (>19 : 1). Following the reduction of the ketone, addition of LiAlH_4_ and heating to reflux afforded full reduction of the lactam to the corresponding tertiary amine. A modified Fieser work up then provided desired amino alcohol **16** in 97% yield. Initially, we found the terminal olefin recalcitrant to both direct and two-step oxidations to the aldehyde due to challenges with olefin isomerization and undesired or poor reactivity. To our delight, olefin cross metathesis using Hoveyda–Grubbs II catalyst of amino alcohol **16** with vinyl boronic acid pinacol ester (**17**) as an aldehyde surrogate smoothly afforded metathesis product **18**. Elaboration of the vinyl boronate was then affected by deprotection and oxidation of the boronic acid, with *in situ* lactolization providing (−)-myrifabral A in 50% yield. X-ray crystallography allowed for the determination of the absolute stereochemistry of the (−)-myrifabral A enantiomeric series. Adaptation of She's conditions for the synthesis of (±)-myrifabral B then provided access to (−)-myrifabral B in 70% yield (Scheme 5).^6*c*^ Interestingly, both (−)-myrifabral B, and (−)-myrifabral A are isolated as oils, whereas racemates of these compounds are isolated as solids.^3*c*,6*c*^ Spectroscopic data obtained were in excellent agreement with the natural compound (see ESI†).

## Conclusions

We have described the first enantioselective total synthesis of (−)-myrifabral A and B. Critical to the success of this strategy was the development of a direct and high yielding one-pot conversion of a Boc-protected amine (**11**) to the glutarimide (**8**). Palladium catalyzed decarboxylative asymmetric allylic alkylation provided the C(10) all-carbon quaternary center in 88% ee, setting the stage for ketone protection, glutarimide reduction, and an exquisitely diastereoselective *N*-acyl iminium ion cyclization. Following ketone and lactam reduction, cross metathesis with vinyl boronic acid pinacol boronate and subsequent boronic acid deprotection and oxidation afforded (−)-myrifabral A. Utilizing previously reported conditions, (−)-myrifabral A was converted to (−)-myrifabral B. This marks the first catalyst-controlled asymmetric synthesis of myrifabral A and B, enabling future biological study of individual enantiomeric series.

## Conflicts of interest

There are no conflicts to declare.

## Supplementary Material

SC-011-D0SC01141J-s001
