# Supplementary material for: Enantioselective total synthesis of (−)-myrifabral A and B
Source: Chem Sci. 2020 Apr 21;11(39):10802–6. doi: 10.1039/d0sc01141j (PMC8162428; doi:10.1039/d0sc01141j)
Supplement: SC-011-D0SC01141J-s001 [file SC-011-D0SC01141J-s001.pdf]

Electronic Supplementary Material (ESI) for Chemical Science.
This journal is © The Royal Society of Chemistry 2020



*Supporting Information for*

# *Enantioselective Total Synthesis of (–)-Myrifabral A and B*

Tyler J. Fulton,[a] Anthony Y. Chen,[a] Michael D. Bartberger,[b] and Brian M. Stoltz*,[a]

[a]*Warren and Katharine Schlinger Laboratory of Chemistry and Chemical Engineering, Division of Chemistry and Chemical Engineering, California Institute of Technology, Pasadena, California 91125, United States*

*stoltz@caltech.edu*

[b]*1200 Pharma LLC, 844 East Green Street, Suite 204, Pasadena, CA, 91101, USA*

*michael.bartberger@1200pharma.com*

## Table of Contents

Materials and Methods ..... S2

List of Abbreviations ..... S2

Synthesis of β-amino ketone **11** ..... S3

Synthesis of Glutarimide **8** ..... S3

Sythesis of Ketone **7** ..... S4

Determination of the Absolute Sterochemistry of Ketone **7** ..... S5

Synthesis of Ethyl Vinyl Ether **14** ..... S8

Synthesis of Tricyclic Lactam **6** ..... S9

Synthesis of Amino Alcohol **16** ..... S10

Synthesis of (–)-Myrifabral A **(4)** ..... S11

Synthesis of (–)-Myrifabral B **(5)** ..... S14

Three Step Synthesis of Glutarimide **8** ..... S17

References ..... S18

NMR and IR Spectra ..... S20

**Materials and Methods**

Unless otherwise stated, reactions were performed in flame-dried glassware under an argon or nitrogen atmosphere using dry, deoxygenated solvents. Solvents were dried by passage through an activated alumina column under argon.[1] Reaction progress was monitored by thin-layer chromatography (TLC) or Agilent 1290 UHPLC-MS. TLC was performed using E. Merck silica gel 60 F254 precoated glass plates (0.25 mm) and visualized by UV fluorescence quenching, *p*-anisaldehyde, oxr $KMnO_4$ staining. Silicycle Silia*Flash*® P60 Academic Silica gel (particle size 40–63 μm) was used for flash chromatography. $^{1}H$ NMR spectra were recorded on Varian Inova 500 MHz and Oxford 600 MHz spectrometers and are reported relative to residual $CHCl_3$ (δ = 7.26 ppm) or TMS (δ = 0.00 ppm). $^{13}C$ NMR spectra were recorded on a Bruker 400 MHz spectrometer (100 MHz) and are reported relative to $CHCl_3$ (δ = 77.16 ppm), $C_6D_6$ (δ = 128.06 ppm) . Data for $^{1}H$ NMR are reported as follows: chemical shift (δ ppm) (multiplicity, coupling constant (Hz), integration). Multiplicities are reported as follows: s = singlet, d = doublet, t = triplet, q = quartet, p = pentet, sept = septuplet, m = multiplet, br s = broad singlet, br d = broad doublet. Data for $^{13}C$ NMR are reported in terms of chemical shifts (δ ppm). IR spectra were obtained by use of a Perkin Elmer Spectrum BXII spectrometer or Nicolet 6700 FTIR spectrometer using thin films deposited on NaCl plates and reported in frequency of absorption ($cm^{-1}$). Optical rotations were measured with a Jasco P-2000 polarimeter operating on the sodium D-line (589 nm), using a 100 mm path-length cell. High resolution mass spectra (HRMS) were obtained from Agilent 6200 Series TOF with an Agilent G1978A Multimode source in electrospray ionization (ESI+), atmospheric pressure chemical ionization (APCI+), or mixed ionization mode (MM: ESI-APCI+). Reagents were purchased from commercial sources and used as received unless otherwise stated.

**List of Abbreviations:**

TLC – thin-layer chromatography
VCD – vibrational circular dichroism
PHOX – phosphinooxazoline

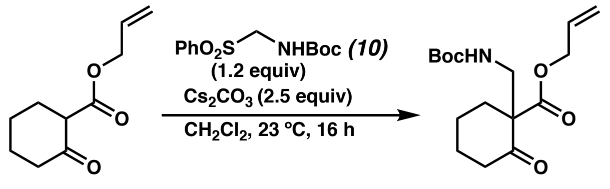


**β-aminoketone (11)**

Compound **11** was prepared as previously described by Stoltz et al.[2] To a stirred solution of β-keto ester **9**[3] (20.0 g, 120.3 mmol, 1.0 equiv) in $CH_2Cl_2$ (600 mL) was added sulfonylmethyl carbamate **10** (39.2 g, 144.4 mmol, 1.2 equiv) in one portion at ambient temperature. After stirring for 5 min, $Cs_2CO_3$ (98.0 g, 300 mmol, 2.5 equiv) was added in one portion. The resulting white suspension was stirred vigorously at 20 ºC. After 16 h, full consumption of starting material was determined by TLC analysis. Saturated aqueous ammonium chloride (300 mL) was added slowly, and the biphasic mixture was stirred at ambient temperature for 40 min and extracted with $CH_2Cl_2$ (3 x 100 mL). The combined organic layers were dried over $Na_2SO_4$, filtered, and concentrated to a viscous, colorless oil. Flash column chromatography ($SiO_2$, 10% EtOAc in hexanes) afforded α-aminomethyl β-keto ester **11** as an amorphous white solid (35.61 g, 114.36 mmol, 95% yield); $^1$H NMR (500 MHz, $CDCl_3$) δ 5.91 (ddt, *J* = 17.2, 10.4, 5.9, 1H), 5.33 (dq, *J* = 17.2, 1.4, 1H), 5.25 (dd, *J* = 10.4, 1.4 Hz, 1H), 5.17 (t, *J* = 5.7 Hz, 1H), 4.63 (d, *J* = 5.8 Hz, 1H), 3.55 (dd, *J* = 13.9, 7.7 Hz, 1H), 3.41 (dd, *J* = 13.9, 5.7 Hz, 1H), 2.63–2.34 (m, 3H), 2.07–1.94 (m, 1H), 1.87–1.75 (m, 1H), 1.74 – 1.53 (m, 4H), 1.41 (s, 9 H); $^{13}$C NMR (100 MHz, $CDCl_3$) δ 208.9, 170.9, 155.9, 131.6, 119.2, 79.4, 66.3, 62.4, 44.3, 40.8, 33.8, 28.4, 27.2, 22.1; IR (Neat Film, NaCl) 3460, 2936, 2869, 1712, 1501, 1451, 1391, 1366, 1315, 1225, 1202, 1170, 1140, 1099, 931, 856 $cm^{-1}$; HRMS (MM:ESI-APCI+) *m/z* calc'd for $C_{16}H_{26}NO_5$ $[M+H]^+$: 312.1805, found 312.1805.

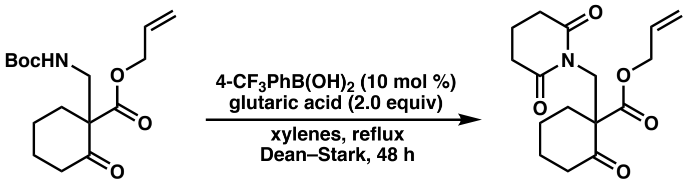


**Glutarimide 8**

A flame-dried 2 L round bottom flask equipped with a Dean–Stark trap, a reflux condenser and a stirring bar was charged with **11** (15.00 g, 48.17 mmol, 1.0 equiv), glutaric acid (12.73 g, 96.35 mmol, 2.0 equiv), 4-$CF_3PhB(OH)_2$ (915.3 mg, 4.82 mmol, 10 mol %), and xylenes (960 mL). The resulting suspension was heated to reflux in a heating mantle with vigorous stirring.

After 48 h, the light orange reaction solution was cooled to 20 ºC and concentrated to provide a crude orange oil. The crude oil was purified by column chromatography (2 → 5% EtOAc in $CH_2Cl_2$) to afford an off white semisolid which was further purified by column chromatography ($SiO_2$, 20→ 40% EtOAc in hexanes) to provide **8** as an amorphous white solid (12.62 g, 41.05 mmol, 85% yield); $^1$H NMR (500 MHz, $CDCl_3$) δ 5.94 (dddd, *J* = 17.3, 10.4, 6.1, 5.6 Hz, 1H), 5.33 (dd, *J* = 17.3, 1.5 Hz, 1H), 5.25 (dd, *J* = 10.4, 1.5 Hz, 1H), 4.66 (ddt, *J* = 13.1, 6.1, 1.2 Hz, 1H), 4.53 (ddt, *J* = 13.1, 5.6, 1.2 Hz, 1H), 4.32 (ABq, $\Delta\delta_{AB}$ = 0.05, $J_{AB}$ = 14.1 Hz, 2H), 2.64 (t, *J* = 6.5 Hz, 4H), 2.48–2.36 (m, 2H), 2.30–2.24 (m, 1H), 2.03–1.97 (m, 1H), 1.94 (p, *J* = 6.6 Hz, 2H), 1.77–1.70 (m, 1H), 1.67–1.49 (m, 3H); $^{13}$C NMR (100 MHz, $CDCl_3$) δ 206.0, 172.8, 169.8, 131.5, 118.6, 66.4, 59.6, 41.9, 40.9, 34.0, 32.8, 27.1, 22.2, 16.7; IR (Neat Film, NaCl) 2942, 1711, 1681, 1426, 1379, 1334, 1265, 1204, 1122, 1098, 1057, 1020 cm$^{-1}$; HRMS (MM:ESI-APCI+) *m/z* calc'd for $C_{16}H_{22}NO_5$ [M+H]$^+$: 308.1492, found 308.1504.

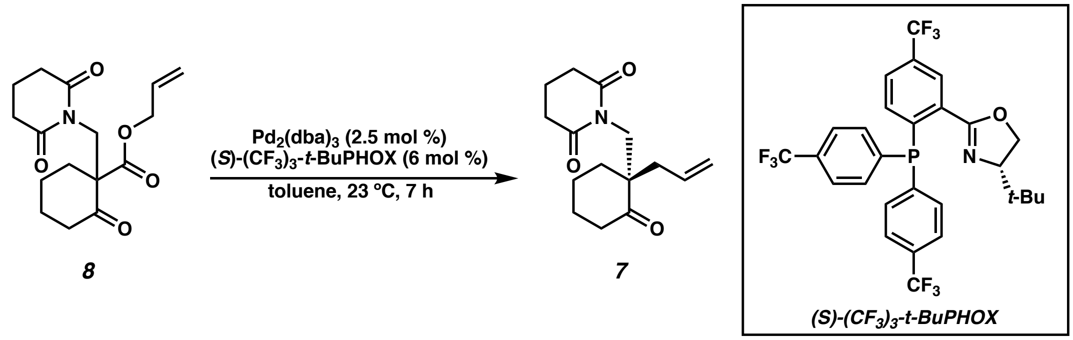


## Ketone 7

An oven-dried 1 L round bottom flask was charged with $Pd_2(dba)_3$ (819.6 mg, 0.895 mmol, 2.75 mol %), (*S*)-$(CF_3)_3$-*t*-BuPHOX (1.1548 g, 1.95 mmol, 6.0 mol %), and a magnetic stirring bar in a $N_2$-filled glovebox. The flask was then charged with toluene (650 mL) and stirred at 24 ºC for 40 min, generating a dark orange/red solution. The preformed catalyst solution was then cannulated into a solution of **8** (10.0 g, 32.54 mmol, 1.0 equiv) dissolved in toluene (325 mL) in a 2 L flame-dried round bottom flask. The resulting dark green solution was stirred at 24 ºC. Full consumption of the starting material was achieved after 7 h, as determined by TLC analysis (25% EtOAc in hexanes). The crude reaction mixture was concentrated and directly purified by column chromatography ($SiO_2$, 10 → 40% EtOAc in hexanes) to yield glutarimide **7** as an off white semi-solid (8.06 g, 30.6 mmol, 94% yield); 88% ee, $[\alpha]_D^{25}$ +32.9 (*c* 1.0, $CHCl_3$); $^1$H NMR (500 MHz, $C_6D_6$) δ 5.67 (dddd, *J* = 16.7, 10.3, 8.4, 5.9 Hz, 1H), 5.04–4.94 (m, 2H), 4.12 (ABq, $\Delta\delta_{AB}$ = 0.22,

$J_{AB}$ = 13.7 Hz, 2H), 2.90 (ddd, $J$ = 15.6, 12.4, 6.2 Hz, 1H), 2.63 (t, $J$ = 6.4 Hz, 4H), 2.38 (ddt, $J$ = 14.2, 5.8, 1.3 Hz, 1H), 2.32 (dt, $J$ = 15.8, 4.4 Hz, 1H), 2.01–1.87 (m, 4H), 1.83–1.68 (m, 4H), 1.64–1.53 (m, 1H); $^{13}$C NMR (100 MHz, $CDCl_3$) δ 213.2, 173.2, 134.7, 118.2, 51.8, 44.2, 39.6, 38.7, 35.3, 33.1, 26.1, 21.2, 16.8; IR (Neat Film, NaCl) 3072, 2937, 2864, 1727, 1701, 1679, 1638, 1430, 1380, 1340, 1319, 1243, 1173, 1120, 1056, 1008, 914, 868, 803 cm$^{-1}$; HRMS (MM:ESI-APCI+) *m/z* calc'd for $C_{15}H_{22}NO_3$ [M+H]$^+$: 264.1594, found 264.1591; SFC Conditions: 35% IPA, 3.5 mL/min, Chiralpak AD-H column, λ = 210 nm, $t_R$ (min): major = 1.28, minor = 1.68.

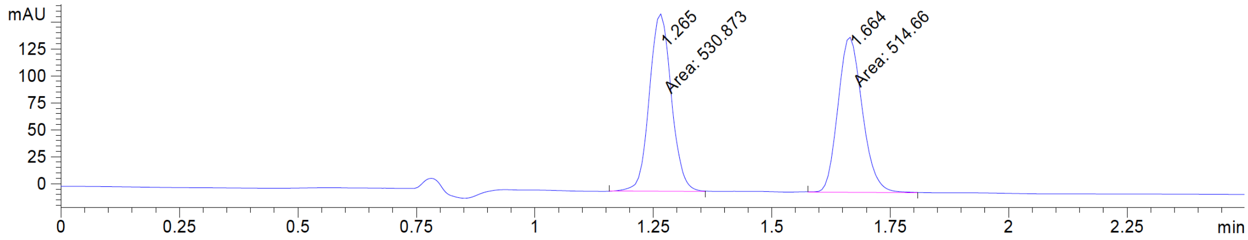


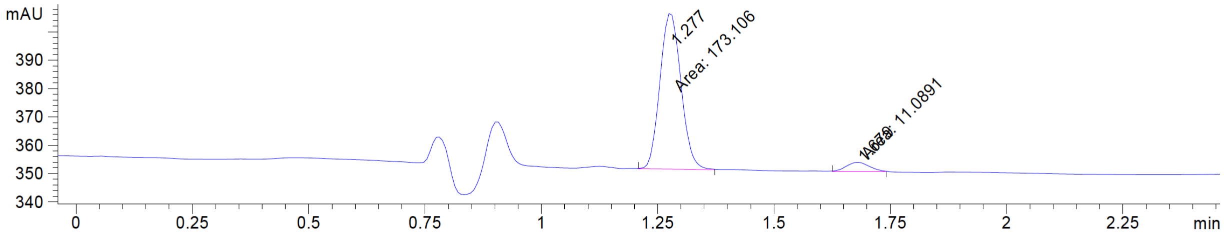


| Peak # | RetTime [min] | Type | Width [min] | Area [mAU*s] | Height [mAU] | Area % |
|---|---|---|---|---|---|---|
| 1 | 1.277 | MM | 0.0520 | 173.10646 | 55.52397 | 93.9797 |
| 2 | 1.679 | MM | 0.0568 | 11.08910 | 3.25302 | 6.0203 |

## **Determination of Absolute Configuration of 7 and *ent*-7.**

### Method 1 – Vibrational Circular Dichroism (VCD)

**Experimental Protocol.** Solutions of compounds **7** and ***ent*-7** (69 mg/mL) were each prepared in $CDCl_3$ and loaded into a front-loading SL-4 cell (International Crystal Laboratories) possessing $BaF_2$ windows and 100 μm path length. Infrared (IR) and VCD spectra were individually acquired on a BioTools ChiralIR-2X VCD spectrometer as sets of 24 one-hour blocks (24 blocks, 3120 scans per block) at 4 cm$^{-1}$ resolution in dual PEM mode. A 15-minute acquisition of neat (–)-α-pinene control (separate 75 μm $BaF_2$ cell) yielded a VCD spectrum in agreement with literature spectra and those previously acquired on the same instrument. IR and VCD spectra were background-corrected using a 30-minute block IR acquisition of the empty instrument chamber under gentle $N_2$ purge, and were solvent corrected using a 12-hour (12 blocks, 3120 scans per

block) IR/VCD acquisition of $CDCl_3$ in the same 100 μm $BaF_2$ cell as used for **7** and ***ent*-7**. The reported spectra represent the result of block averaging.

**Computational Protocol**. The arbitrarily chosen (*R*) enantiomer of compound **7** was subjected to an initial exhaustive stochastic molecular mechanics-based conformational search (MMFF94 force field, 0.06 Å geometric RMSD cutoff, and 30 kcal/mol energy window) as implemented in MOE 2019.0102 (Chemical Computing Group, Montreal, CA). All conformers retained the *(R)* configuration and were subjected to geometry optimization, harmonic frequency calculation, and VCD rotational strength evaluation using density functional theory. Initial quantum mechanical calculations utilized the B3LYP functional, small 6-31G* basis, and IEFPCM model (chloroform solvent) as an initial filter. This was followed by subsequent treatment using the B3PW91 functional, cc-pVTZ basis, and implicit IEFPCM chloroform solvation model on all IEFPCM-B3LYP/6-31G* conformers below 5 kcal/mol, reusing the exact Hessian of the latter to facilitate optimization at the higher level of theory. All calculations were performed with the *Gaussian 16* program system (Rev. C.01; Frisch *et al*., Gaussian, Inc., Wallingford, CT). Resultant IEFPCM-B3PW91/cc-pVTZ harmonic frequencies were scaled by 0.98. All structurally unique conformers possessing all positive Hessian eigenvalues were Boltzmann weighted by relative free energy at 298.15 K. The predicted IR and VCD frequencies and intensities of the retained conformers were convolved using Lorentzian line shapes ($\gamma = 4\ cm^{-1}$) and summed using the respective Boltzmann weights to yield the final predicted IR and VCD spectra. The predicted VCD spectrum of the *(S)* enantiomer was generated by inversion of sign. From the outstanding agreement between the theoretical and measured IR and VCD spectra across the entire useful range of the spectrum (900–1500 $cm^{-1}$; regions **A**–**J** below) along with support of this assignment using the directly predicted versus measured optical rotations (see Method 2) the absolute configurations of species **7** and ***ent*-7** were established as *(R)* and *(S)*, respectively.

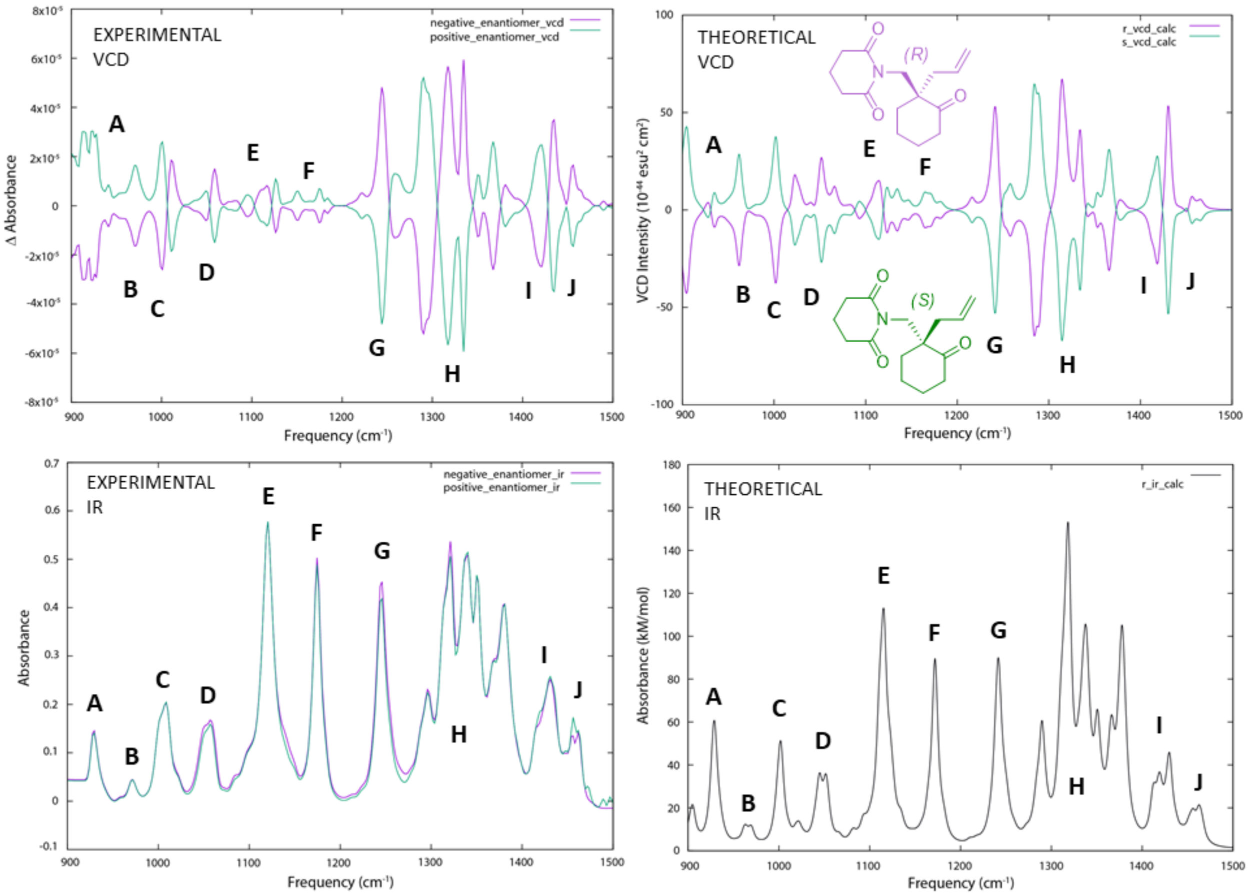


Experimental (left) and computed (right) IR and VCD spectra of **7** and ***ent*-7.**

**Method 2 – Optical Rotation (OR)**

**Computational Protocol**. The ensemble of unique IEFPCM-B3PW91/cc-pVTZ conformers of ***(R)*-7** generated in Method 1 above were subjected to optical rotation calculation at 589.0 nm using the B3LYP hybrid density functional, the large and diffuse 6-311++G(2df,2pd) basis set, and the IEFPCM implicit chloroform solvent model. The computed IEFPCM-B3LYP/6-311++G(2df,2pd) optical rotations (weighted by IEFPCM-B3PW91/cc-pVTZ free energies at 298.15 K) along with those resulting from alternatively weighting by either the IEFPCM-B3PW91/cc-pVTZ total energies or IEFPCM-B3LYP/6-311++G(2df,2pd)//IEFPCM-B3PW91/cc-pVTZ total energies are reported in (a)–(b) below. From comparison of the theoretically calculated and measured optical rotations (for which reasonably good agreement in magnitude was found to exist) the respective VCD-based AC assignments of *(R)* and *(S)* for **7** and ***ent*-7** were further supported by those from the separate OR-based methodology. The individual

relative energies, free energies, and optical rotation signatures of each conformer of ***(R)*-7** are provided in the accompanying Microsoft Excel file.

(a) 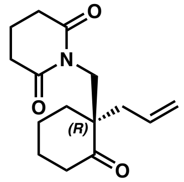


Predicted optical rotation, weighted by IEFPCM-B3PW91/cc-pVTZ free energies: **–47.5°**

Predicted optical rotations, weighted by IEFPCM-B3PW91/cc-pVTZ total energies: **–45.5°**

Predicted optical rotations, weighted by IEFPCM-B3LYP/6-311++G(2df,2pd)//IEFPCM-B3PW91/cc-pVTZ total energies: **–45.8°**

∴

(b) 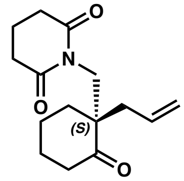


Predicted optical rotation, weighted by IEFPCM-B3PW91/cc-pVTZ free energies: **+47.5°**

Predicted optical rotations, weighted by IEFPCM-B3PW91/cc-pVTZ total energies: **+45.5°**

Predicted optical rotations, weighted by IEFPCM-B3LYP/6-311++G(2df,2pd)//IEFPCM-B3PW91/cc-pVTZ total energies: **+45.8°**

Measured optical rotation: ($CHCl_3$ solvent, 25 °C, c = 10.0 mg/mL, 10 cm pathlength, 88% ee)

**7: + 32.9**

***ent*-7: –28.9**

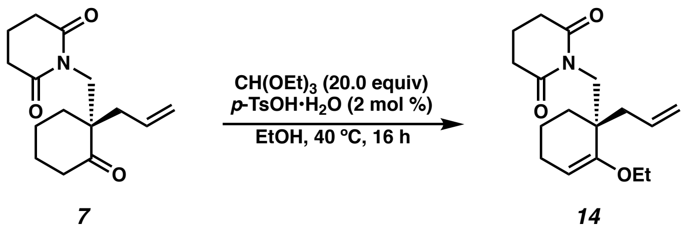


**Ethyl vinyl ether 14**

A flame-dried 250 mL round bottom flask was charged with **7** (3.00 g, 11.4 mmol, 1.0 equiv), EtOH (114 mL), *p*-TsOH•$H_2O$ (43.4 mg, 0.228 mmol, 0.02 equiv), and $CH(OEt)_3$ (38.0

mL, 228 mmol, 20.0 equiv). The resulting clear, colorless solution was heated in a 40 ºC heating block for 16 h, after which time complete conversion was observed by TLC analysis (40% EtOAc in hexanes). The reaction mixture was concentrated under reduced pressure and the resulting colorless oil was dissolved in EtOAc (50 mL) and poured into a separatory funnel containing saturated aqueous $NaHCO_3$ (50 mL). The layers were separated and the aqueous layer was extracted with EtOAc (2 x 100 mL). The combined organics were washed with brine (25 mL), dried over $Na_2SO_4$, filtered, and concentrated. Flash column chromatography ($SiO_2$, 30% EtOAc in hexanes) provided **14** as a colorless oil (3.0326 g, 10.41 mmol, 91% yield); $[\alpha]_D^{25}$ –72.6 (*c* 1.0, $CHCl_3$); $^1H$ NMR (500 MHz, $C_6D_6$) δ 5.90 (dddd, *J* = 17.3, 10.0, 9.2, 5.3 Hz, 1H), 5.18–5.01 (m, 2H), 4.66–4.54 (m, 1H), 4.35 (ABq, $\Delta\delta_{AB}$ = 0.23, $J_{AB}$ = 13.1 Hz, 2H), 3.55–3.31 (m, 2H), 2.95 (ddt, *J* = 13.4, 5.1, 1.3 Hz, 1H), 2.14–1.81 (m, 8H), 1.77–1.64 (m, 1H), 1.64–1.50 (m, 2H), 1.10 (t, *J* = 7.0 Hz, 3H), 1.06 – 0.91 (m, 2H); $^{13}C$ NMR (100 MHz, $C_6D_6$) δ 172.2, 156.5, 136.4, 117.1, 96.6, 61.8, 45.1, 42.7, 41.4, 33.2, 31.8, 24.5, 19.5, 16.8, 14.8; IR (Neat Film, NaCl) 3393, 3071, 2974, 2935, 2876, 2839, 1730, 1682, 1430, 1359, 1341, 1275, 1240, 1220, 1176, 1158, 1138, 1113, 1057, 1046, 1002, 930, 912, 879, 846, 816, 787, 745, 698 $cm^{-1}$; HRMS (MM:ESI-APCI+) *m/z* calc'd for $C_{17}H_{26}NO_3$ $[M+H]^+$: 292.1907, found 292.1910.

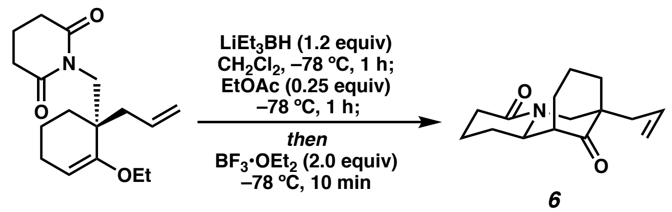


**14** **6**

Tricyclic lactam **6**

A flame-dried 250 mL round bottom flask was charged with **14** (2.50 g, 8.58 mmol, 1.0 equiv) and $CH_2Cl_2$ (86 mL). The resulting clear, colorless solution was cooled in a –78 ºC bath. After 15 min, $LiEt_3BH$ in (9.44 mL, 1.0 M in THF, 9.44 mmol, 1.1 equiv) was added dropwise over 5 min. After 30 min, an additional portion of $LiEt_3BH$ (360 µL, 0.360 mmol, 0.042 equiv) was added. After 10 min, an additional portion of $LiEt_3BH$ (300 µL, 0.300 mmol, 0.035 equiv) was added. After stirring for 10 min, the reaction was complete by TLC analysis (40% EtOAc in hexanes). EtOAc (210 µL, 2.15 mmol, 0.25 equiv) was added and the reaction was allowed to stir at –78 ºC. After 1 h, $BF_3$•$OEt_2$ (2.11 mL, 17.2 mmol, 2.0 equiv) was added dropwise over 5 min. After 15 min, the reaction was complete by LC/MS analysis. The reaction mixture was quenched with $H_2O$ (60 mL), warmed to 20 ºC, and transferred to a separatory funnel with $CH_2Cl_2$ (10 mL).

The layers were separated and the aqueous was extracted with $CH_2Cl_2$ (3 x 25 mL). The combined organics were dried over $Na_2SO_4$, filtered, and concentrated to afford a pale yellow oil. Purification by column chromatography ($SiO_2$, 25% acetone in hexanes) yielded **6** as a white semi-solid (1.8841 g, 7.62 mmol, 89% yield); $[\alpha]_D^{25}$ –22.6 (*c* 1.0, $CHCl_3$); $^1H$ NMR (400 MHz, $C_6D_6$) δ 5.91 (ddt, *J* = 17.4, 10.2, 7.5 Hz, 1H), 5.06–4.93 (m, 3H), 2.97 (dtd, *J* = 10.6, 5.5, 2.8 Hz, 1H), 2.49 (dd, *J* = 13.7, 2.4 Hz, 1H), 2.29 (ddt, *J* = 17.1, 4.8, 2.6 Hz, 1H), 2.18 (dq, *J* = 7.4, 1.3 Hz, 2H), 1.93 (dq, *J* = 4.9, 2.7 Hz, 1H), 1.91–1.72 (m, 4H), 1.48–1.30 (m, 2H), 1.25 – 1.15 (m, 1H), 1.15–1.05 (m, 1H), 1.04 – 0.95 (m, 2H), 0.94–0.85 (m, 1H); $^{13}C$ NMR (100 MHz, $C_6D_6$) δ 213.8, 168.5, 134.1, 118.3, 59.9, 52.8, 51.6, 49.1, 39.6, 39.2, 33.3, 28.9, 26.7, 20.5, 19.7; IR (Neat Film, NaCl) 3074, 2937, 2870, 1716, 1644, 1460, 1440, 1416, 1346, 1264, 1235, 1166, 1120, 1064, 994, 958, 916 $cm^{-1}$; HRMS (MM:ESI-APCI+) *m/z* calc'd for $C_{15}H_{21}NO_2$ $[M+H]^+$: 248.1645, found 248.1653.

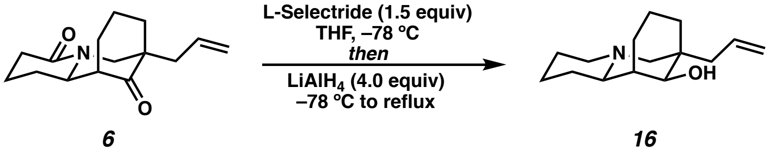


Amino alcohol **16**

A two-necked 250 mL round bottom flask equipped with a reflux condenser, septum, and stir bar was charged with **6** (905 mg, 3.66 mmol, 1.0 equiv) and THF (73 mL). A flame-dried 25 mL conical flask under $N_2$ was charged with L-Selectride (5.90 mL, 1.0 M in THF, 5.90 mmol, 1.6 equiv). Both flasks were cooled to –78 ºC for 30 min, after which time the L-Selectride solution was slowly transferred to the flask containing **6** via syringe over 20 min via positive pressure cannulation, resulting in the formation of an opaque white reaction mixture. After 30 min, the septum was exchanged for an oven-dried glass stopper and $LiAlH_4$ (555.6 mg, 14.6 mmol, 4.0 equiv) was added in a single portion to the reaction mixture. The reaction was then removed from the cooling bath and allowed to reach 20 ºC, after which time the flask was immersed in a 75 ºC oil bath. After refluxing for 9 h, complete conversion to **16** was observed by LC/MS and TLC analysis. The reaction mixture was diluted with $Et_2O$ (50 mL) and cooled in an ice/water bath. After 10 min, the reaction was slowly quenched with dropwise addition of $H_2O$ (800 μL) over 10 min, followed by the addition of aqueous NaOH (4.0 mL of a 2.5 M solution), and $H_2O_2$ (4.0 mL of a 30% solution). The resulting gray suspension was then stirred vigorously for 30 min before being filtered through a pad of celite (5 x 5 cm), washing with EtOAc (3 x 75 mL). The filtrate

was then transferred to a separatory funnel and washed with $H_2O$ (60 mL) and brine (60 mL). The combined aqueous layers were extracted with EtOAc (3 x 80 mL). The combined organics were then dried over $Na_2SO_4$, filtered, and concentrated to a pale yellow oil. Purification by column chromatography ($SiO_2$, 25% EtOAc in hexanes with 1% $Et_3N$) afforded **16** as a colorless, viscous oil which slowly turns red with exposure to air (833.1 mg, 3.54 mmol, 97% yield); $[\alpha]_D^{25}$ –13.4 (*c* 1.0, $CHCl_3$); $^1$H NMR (500 MHz, $C_6D_6$) δ 5.83 (dddd, *J* = 15.1, 11.2, 8.3, 7.6 Hz, 1H), 5.04 (app t, *J* = 1.2 Hz, 1H), 5.02–4.99 (m, 1H), 3.25 (m, 1H), 2.78–2.60 (m, 2H), 2.55 (d, *J* = 11.3 Hz, 1H), 2.08–1.74 (m, 6H), 1.68–1.37 (m, 9H), 1.32–1.03 (m, 3H); $^{13}$C NMR (100 MHz, $C_6D_6$) δ 134.8, 116.8, 75.4, 65.5, 65.4, 56.4, 43.3, 41.8, 37.8, 30.5, 30.0, 26.1, 24.7, 20.9, 20.0; IR (Neat Film, NaCl) 3404 (br), 3072, 2931, 2856, 2797, 2759, 1638, 1463, 1442, 1375, 1336, 1271, 1223, 1198, 1182, 1124, 1106, 1044, 995, 958, 947, 912, 844, 813, 768, 714, 678, 635 cm$^{-1}$; HRMS (MM:ESI-APCI+) *m/z* calc'd for $C_{15}H_{26}NO$ $[M+H]^+$: 236.2009, found 236.2012.

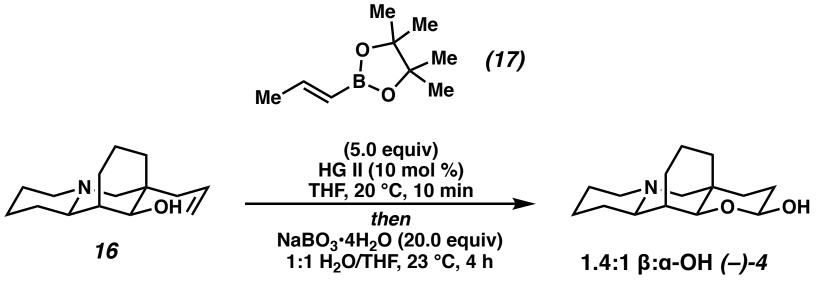


(–)-α,β-myrifabral A (**4**)

In a nitrogen-filled glovebox, an oven-dried 25 mL round bottom flask was charged with amino alcohol **16** (100.0 mg, 0.424 mmol, 1.0 equiv), pinacol boronate **17** (356.2 mg, 2.12 mmol, 5.0 equiv), and a Teflon-coated stir bar. The flask was sealed with a septum, removed from the glovebox, and placed under an atmosphere of nitrogen. To the flask was added THF (2.2 mL) to provide a clear, colorless solution. Hoveyda–Grubbs II catalyst (26.6 mg, 0.0424 mmol, 10 mol %) was then added rapidly in a single portion, and the flask was subjected to vacuum until the green solution began to bubble. The dark green reaction was allowed to stir for 10 min under a static vacuum, at which point the flask was backfilled with nitrogen and an aliquot was analyzed by LC/MS, indicating full conversion of amino alcohol **16** to the putative cross metathesis product. *Note: Typically, the reaction solution rapidly turns dark brown/black when the flask is backfilled with nitrogen*. The metathesis catalyst was quenched with the addition of ethyl vinyl ether (40 μL) at 20 °C. After stirring for 30 min at 20 °C, deionized water (2.2 mL) and $NaBO_3•4H_2O$ (1.30 g, 8.48 mmol, 20.0 equiv) were added and the resulting black, biphasic suspension was stirred rapidly

at 20 °C. After 4 h, full conversion of the intermediate cross metathesis product was observed by LC/MS analysis. The reaction mixture was poured into a separatory funnel with EtOAc (5 mL) and $H_2O$ (10 mL). The layers were separated and the aqueous layer was extracted 3 x 5 mL EtOAc. The combined organics were dried over $Na_2SO_4$, filtered, and concentrated to yield a dark brown oil. Purification by column chromatography ($SiO_2$, 0→50% EtOAc in hexanes with 2% $Et_3N$) yielded (–)-myrifabral A (**4**) as a 1.4:1 mixture of β:α-OH epimers as a viscous yellow oil (90.1 mg, 0.358 mmol, 85% yield). Note: racemic samples of this compound are isolated as a colorless solid, in accordance with previous reports.[4,5] Due to the complicated overlap of β and α-OH epimers of (–)-4, the $^{1}H$ NMR spectral data are reported with raw integration values; $[\alpha]_D^{25}$ –41.4 (*c* 1.0, $CHCl_3$); $^{1}H$ NMR (600 MHz, Pyridine-$d_5$) δ 8.36 (s, 1.00), 7.70 (s, 0.72), 5.68 (d, $J$ = 3.7 Hz, 0.82), 5.10 (d, $J$ = 9.6 Hz, 1.19), 4.19 (d, $J$ = 3.4 Hz, 0.89), 3.21 (d, $J$ = 3.6 Hz, 1.17), 2.80–2.64 (m, 4.30), 2.54 (t, $J$ = 10.7 Hz, 2.16), 2.30–2.19 (m, 2.22), 2.10 – 1.88 (m, 7.28), 1.88 – 1.80 (m, 2.19), 1.78–1.72 (d, $J$ = 11.9 Hz, 3.43), 1.68–1.59 (m, 8.86), 1.57–1.40 (m, 8.86), 1.27 – 1.05 (m, 10.40); $^{13}C$ NMR (100 MHz, Pyridine-$d_5$) δ 98.3, 92.3, 80.7, 72.5, 69.6, 69.1, 66.8, 66.4, 57.2, 57.2, 40.4, 40.2, 34.4, 33.1, 32.6, 30.7, 30.7, 30.6, 29.6, 29.6, 28.7, 27.5, 26.9, 26.9, 25.5, 25.5, 21.4, 21.3, 21.3, 21.2; IR (Neat Film, NaCl) 3381 (br), 3054, 2933, 2851, 2796, 2756, 2728, 2253, 1714, 1562, 1549, 1540, 1462, 1456, 1444, 1396, 1374, 1336, 1298, 1277, 1243, 1209, 1189, 1124, 1106, 1077, 1057, 1009, 998, 961, 949, 928, 917, 902, 886, 870, 856, 840, 826, 762, 739, 704, 673 $cm^{-1}$, HRMS (MM:ESI-APCI+) *m/z* calc'd for $C_{15}H_{26}NO_2$ $[M+H]^+$: 252.1958, found 252.1960.

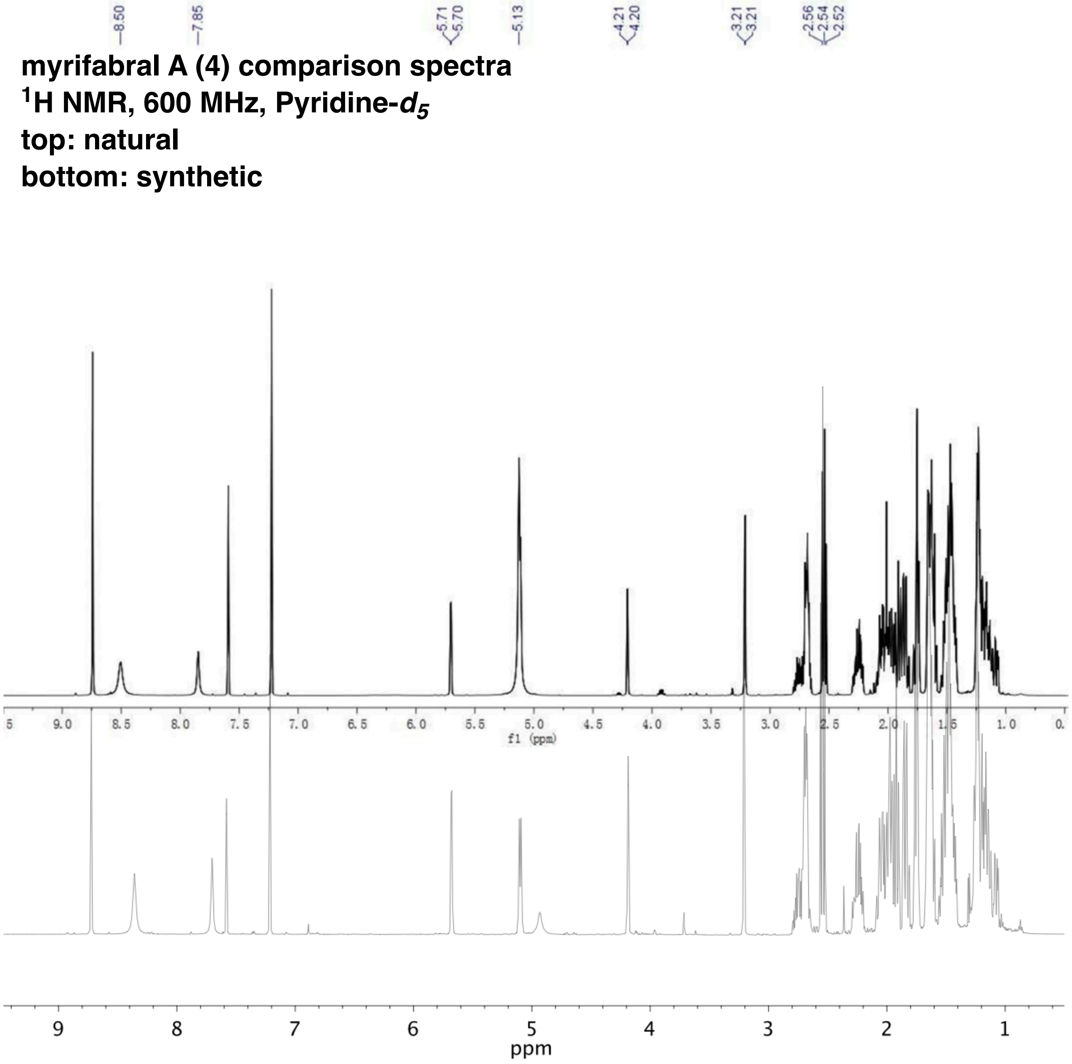
8.50
7.85
5.71
5.70
5.13
4.21
4.20
3.21
3.21
2.56
2.54
2.52
**myrifabral A (4) comparison spectra**
**$^1$H NMR, 600 MHz, Pyridine-$d_5$**
**top: natural**
**bottom: synthetic**
f1 (ppm)
ppm

**myrifabral A (4) comparison spectra**
**$^{13}C$ NMR, 100 MHz, Pyridine-$d_5$**
**top: natural**
**bottom: synthetic**

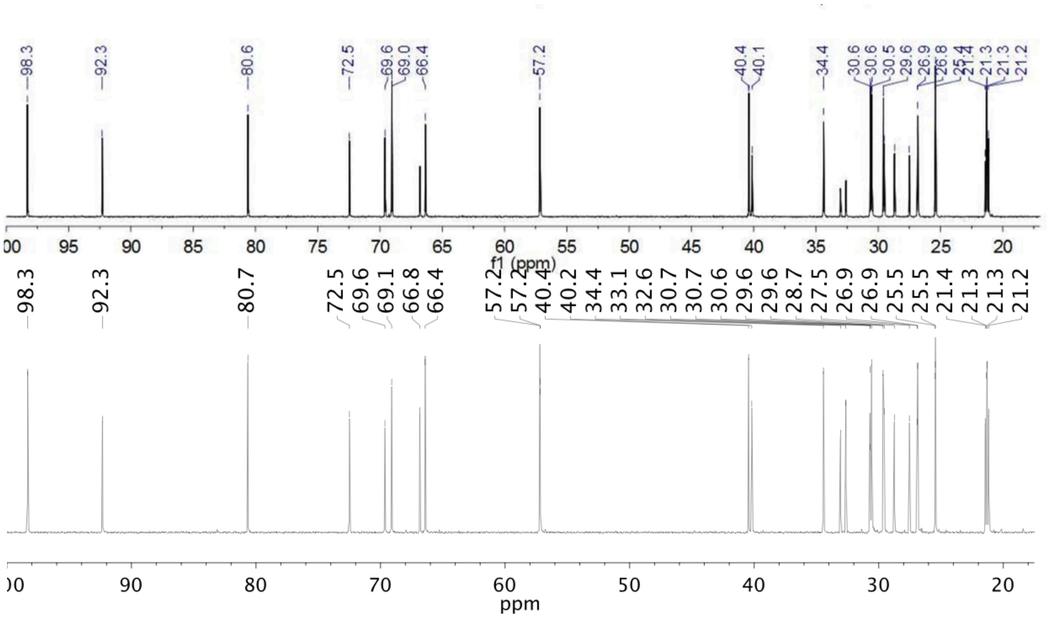


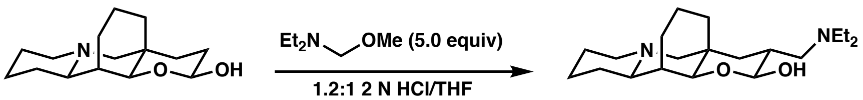


***1.4:1 β:α-OH (–)-4*** **1.6:1 α:β-OH *(–)-5***

(–)-α,β-myrifabral B (**5**)

The following procedure was adapted from Song's total synthesis of (±)-myrifabral B.[5] To a one dram vial with a stir bar was added (–)-**4** (50.0 mg, 0.199 mmol, 1.0 equiv), THF (220 μL), 2 N aqueous HCl (250 μL), and *N*-ethyl-*N*-(methoxymethyl)ethanamine (117.2 mg, 0.995 mmol, 5.0 equiv) to provide a colorless, biphasic reaction mixture. The vial was sealed with a Teflon-lined cap and heated in a vial well at 80 °C with rapid stirring. After 4 h, the reaction was complete by LC/MS analysis. The vial was cooled to 20 °C, then the biphasic reaction mixture was poured into a separatory funnel with EtOAc (5 mL), and saturated aqueous $NaHCO_3$ (10 mL). The layers were separated and the aqueous layer was extracted with EtOAc (3 x 5 mL). The combined organics were dried over $Na_2SO_4$, filtered, and concentrated to a brown/yellow oil. Purification by column chromatography ($SiO_2$, 20→60% EtOAc in hexanes with 2% $Et_3N$) yielded (–)-myrifabral B (**5**) as a 1.6:1 mixture of β:α-OH epimers as a viscous yellow oil (29.5 mg, 0.0877

mmol, 44% yield). Note: racemic samples of this compound are isolated as a colorless solid, in accordance with previous reports.[3,4] Due to the complicated overlap of β and α-OH epimers of (–)-5, the $^{1}$H NMR spectral data are reported with raw integration values; $[\alpha]_D^{25}$ –37.5 (*c* 1.0, $CHCl_3$); $^{1}$H NMR (600 MHz, Pyridine-$d_5$) δ 5.72 (d, *J* = 3.0 Hz, 1.00), 4.82 (d, *J* = 8.2 Hz, 1.67), 4.22 (d, *J* = 3.4 Hz, 1.01), 4.22 (d, *J* = 3.4 Hz, 1.40), 2.83–2.74 (m, 2.40), 2.73–2.68 (m, 3.86), 2.66 (dd, *J* = 12.7, 7.9 Hz, 1.75), 2.60 (dd, *J* = 10.9, 7.2 Hz, 2.92), 2.57–2.47 (m, 7.18), 2.45–2.33 (m, 6.41), 2.28 (tdd, *J* = 13.1, 6.6, 2.4 Hz, 1.64), 2.21 (dd, *J* = 12.8, 6.1 Hz, 1.61), 2.15–2.01 (m, 4.23), 2.01–1.93 (m, 4.26), 1.83–1.78 (m, 3.00), 1.73–1.60 (m, 9.07), 1.58–1.43 (m, 11.78), 1.39 (dd, *J* = 13.3, 4.4 Hz, 1.56), 1.34–1.28 (m, 3.61 ), 1.25 (dt, *J* = 13.0, 3.1 Hz, 2.86), 1.21 – 1.09 (m, 3.00), 1.02 (t, *J* = 7.1 Hz, 5.89), 0.99 (t, *J* = 7.1 Hz, 8.22), 0.85 (t, *J* = 12.9 Hz, 1.39); $^{13}$C NMR (100 MHz, Pyridine-$d_5$) δ 103.1, 94.2, 80.4, 72.6, 69.8, 69.0, 66.9, 66.4, 57.2, 57.2, 57.1, 56.8, 48.5, 48.0, 40.1, 40.0, 39.1, 37.3, 35.2, 35.2, 33.8, 33.8, 30.7, 30.7, 30.5, 29.8, 26.9, 26.8, 25.5, 25.4, 21.6, 21.4, 21.3, 21.3, 12.9, 12.4; IR (Neat Film, NaCl) 3076 (br), 2966, 2933, 2851, 2801, 2757, 2728, 2251, 1722, 1692, 1557, 1462, 1444, 1375, 1346, 1297, 1276, 1267, 1243, 1205, 1196, 1178, 1144, 1124, 1104, 1089, 1076, 1059, 1037, 967, 918, 904, 885, 813, 860, 833, 753, 715, 666, 616 $cm^{-1}$, HRMS (FAB+) *m/z* calc'd for $C_{20}H_{37}N_2O_2$ $[M+H]^+$: 337.2855, found 337.2857.

**myrifabral B (5) comparison spectra**
**$^1$H NMR, 600 MHz, Pyridine-$d_5$**
**top: natural**
**bottom: synthetic**

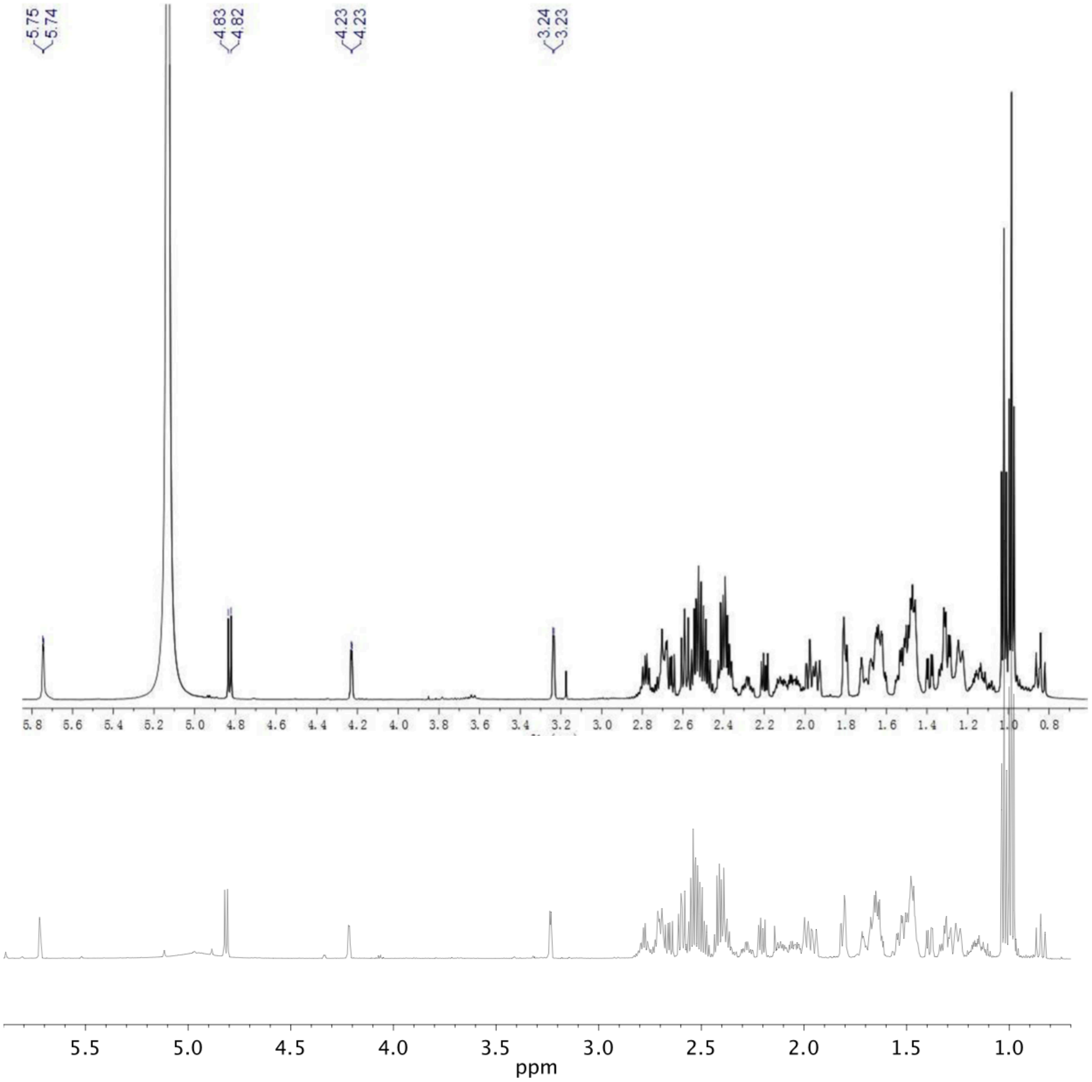

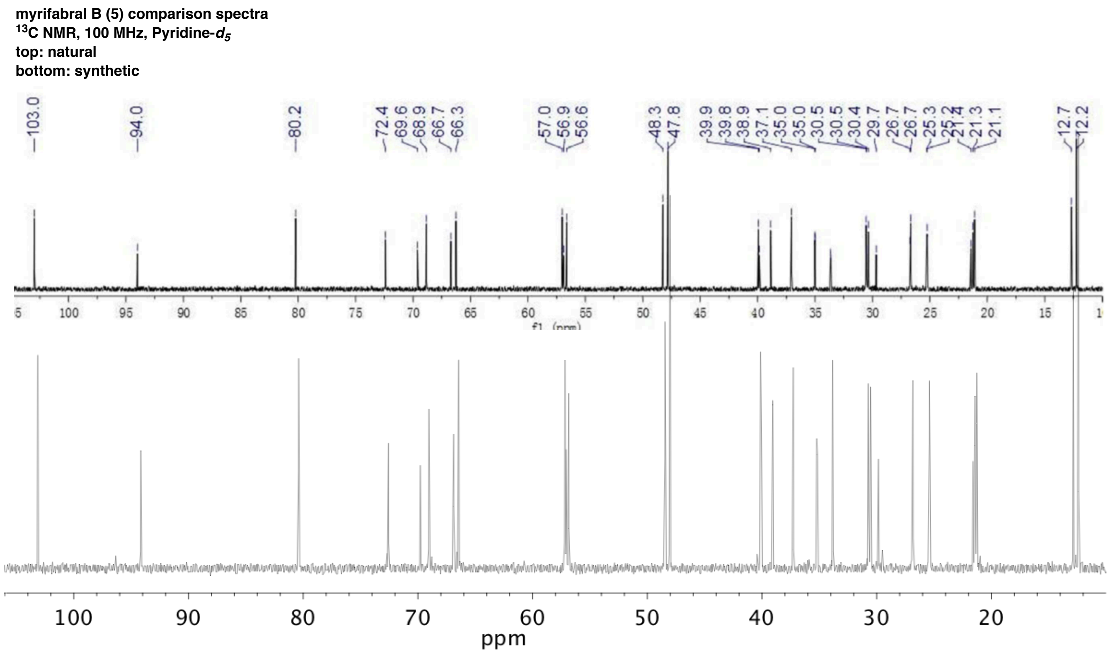


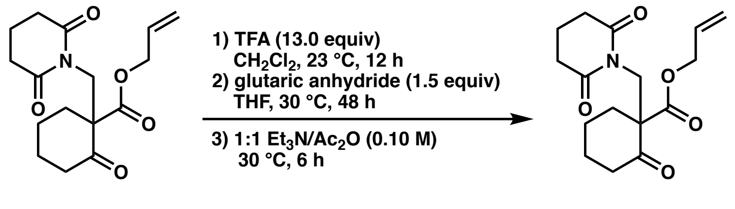


***11*** ***8***

Three Step Synthesis of Glutarimide **8**

The following procedure was adapted from reports from Toda and Pérez-Castells.[6] A flame-dried 250-mL round bottom flask equipped with a Teflon coated stir bar was charged with **11** (1.2935g, 4.15 mmol, 1.0 equiv), $CH_2Cl_2$ (38 mL), and TFA (4.2 mL) under an argon atmosphere. The resulting clear, colorless solution was stirred at 21 °C for 12 h, after which time consumption of **11** was observed by LC/MS analysis. The reaction mixture was concentrated under reduced pressure then dried under high vacuum to yield a dark brown oil. The flask was

then backfilled with argon. To the flask was then added THF (41.5 mL) and glutaric anhydride (710.8 mg, 6.23 mmol, 1.50 equiv). The resulting pale yellow solution was stirred rapidly at 30 °C in a pre-heated oil bath. After 48 h, the reaction mixture was concentrated under reduced pressure to a yellow/orange oil. The flask was then purged and evacuated with argon (3 cycles). To the flash was then added $Ac_2O$ (21 mL) and $Et_3N$ (21 mL), resulting in a golden yellow solution which was stirred in a pre-heated 30 °C oil bath. After 6 h, the golden yellow reaction mixture was concentrated under reduced pressure, diluted with EtOAc (100 mL), and transferred to a separatory funnel. The organic layer was then washed with 1 N HCl (2 x 80 mL), sat. aq. $NaHCO_3$ (1 x 80 mL), and brine (1 x 80 mL). The organic layer was then dried over $Na_2SO_4$, filtered, and concentrated to an orange/yellow oil. The oil was then dissolved in EtOAc (100 mL) and washed with $H_2O$ (5 x 50 mL) to remove excess $Ac_2O$ and glutaric anhydride. Purification by column chromatography (50% EtOAc in hexanes) yielded glutarimide **8** as a semi-crystalline white solid (462.0 mg, 1.50 mmol, 36% yield). Characterization data are provided above (see S4).

**References**

(1) A. M. Pangborn, M. A. Giardello, R. H. Grubbs, R. K. Rosen, F. J. Timmers, Safe and Convinient Procedure for Solvent Purification. *Organometallics* **1996**, *15*, 1518–1520.

(2) Y. Numajiri, B. P. Pritchett, K. Chiyoda, B.M. Stoltz, Enantioselective Synthesis of α-Quaternary Mannich Adducts by Palladium-Catalyzed Allylic Alkylation: Total Synthesis of (+)-Sibirinine. *J. Am. Chem. Soc.* **2015**, *137*, 1040–1043.

(3) J. T. Mohr, M. R. Krout, B. M. Stoltz, Preparation of *(S)*-2-allyl-2-methylcyclohexanaone. *Org. Synth.* **2009**, *86*, 194–211.

(4) M.-M. Cao, Y. Zhang, X.-H. Li, Z.-G. Peng, J.-D. Jiang, Y.-C. Gu, Y.-T. Di, X.-N. Li, D.-Z. Chen, C.-F. Xia, H.-P. He, S.-L. Li, X.-J. Hao, Cyclohexane-Fused Octahydroquinolizine Alkaloids from *Myrioneuron faberi* with Activity against Hepatitis C Virus. *J. Org. Chem.* **2014**, *79*, 7945–7950.

(5) D. Song, Z. Wang, R. Mei, W. Zhang, D. Ma, D. Xu, X. Xie, X. She, Short and Scalable Total Synthesis of Myrioneuron Alkaloids (±)-α,β-Myrifabral A and B. *Org. Lett.* **2016**, *18*, 669–671.

(6) (a) Y. Kawanaka, K. Kobayashi, S. Kusuda, T. Tatsumi, M. Murota, T. Nishiyama, K. Hisaichi, A. Fujii, K. Hirai, M. Naka, M. Komeno, Y. Odagaki, H. Nakai, M. Toda, Design and Synthesis of Orally Bioavailable Inhibitors of Inducible Nitric Oxide Synthase. Identification of 2-Azabicyclo[4.1.0]heptan-3-imines. *Biorg. Med. Chem.* **2003**, *11*, 1723–

1743. (b) I. S. del Villar, A. Gradillas, J. Pérez-Castells, Synthesis of 2-Azabicyclo[4.1.0]heptanes through Stereoselective Cyclopropanation Reactions. *Eur. J. Org. Chem.* **2010**, 5850–5862.

## NMR and IR Spectra

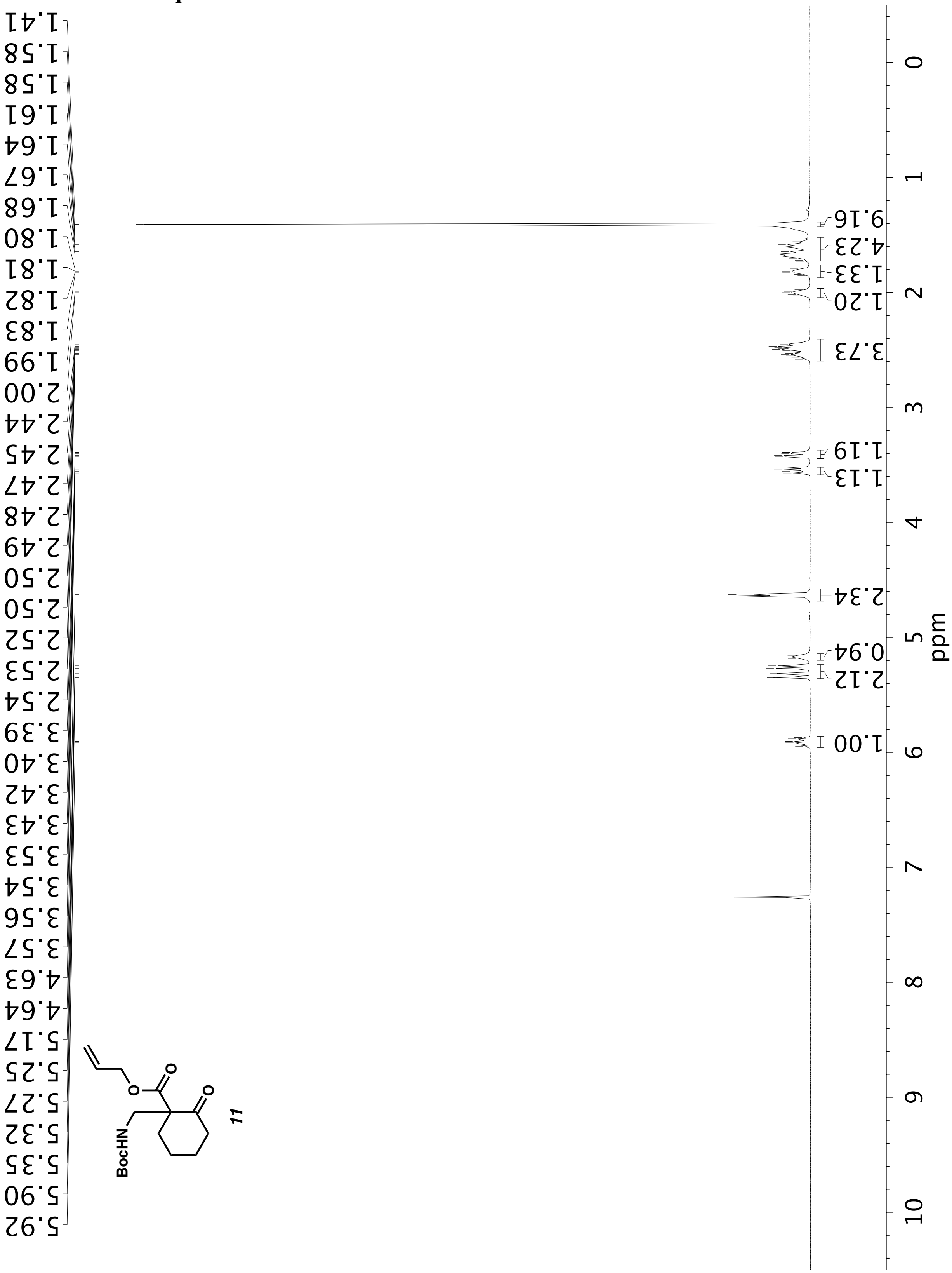


$^1H$ NMR (500 MHz, $CDCl_3$) of compound **11**.

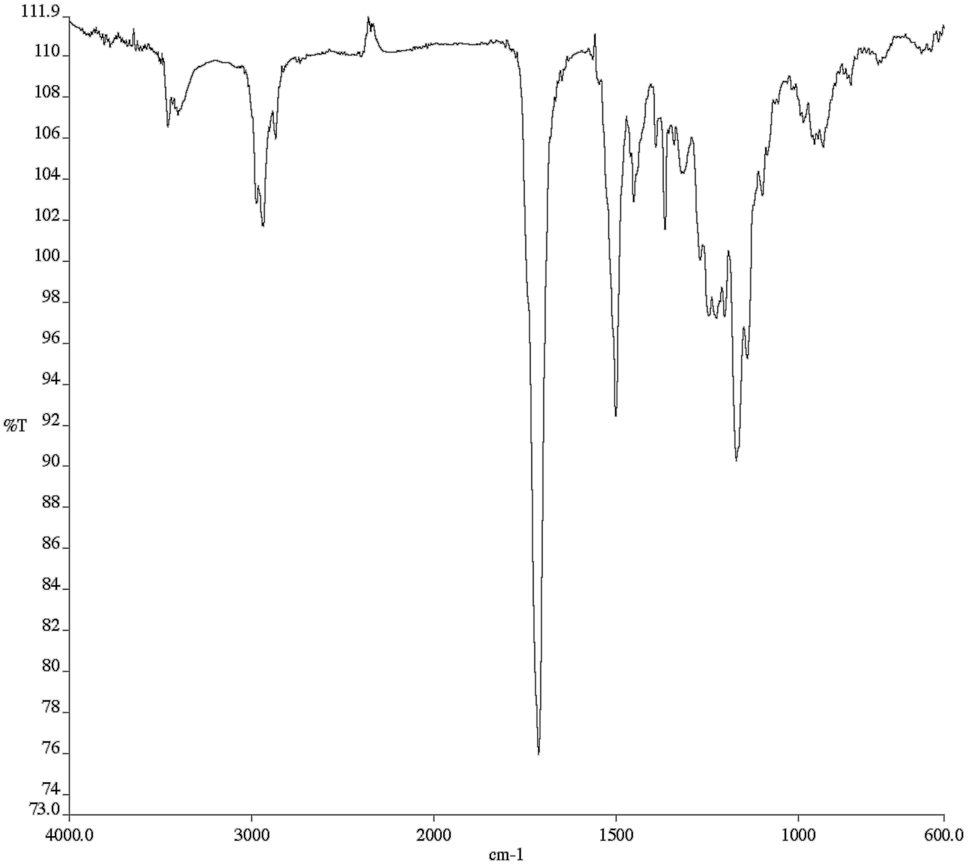


Infrared spectrum (Thin Film, NaCl) of compound **11**.

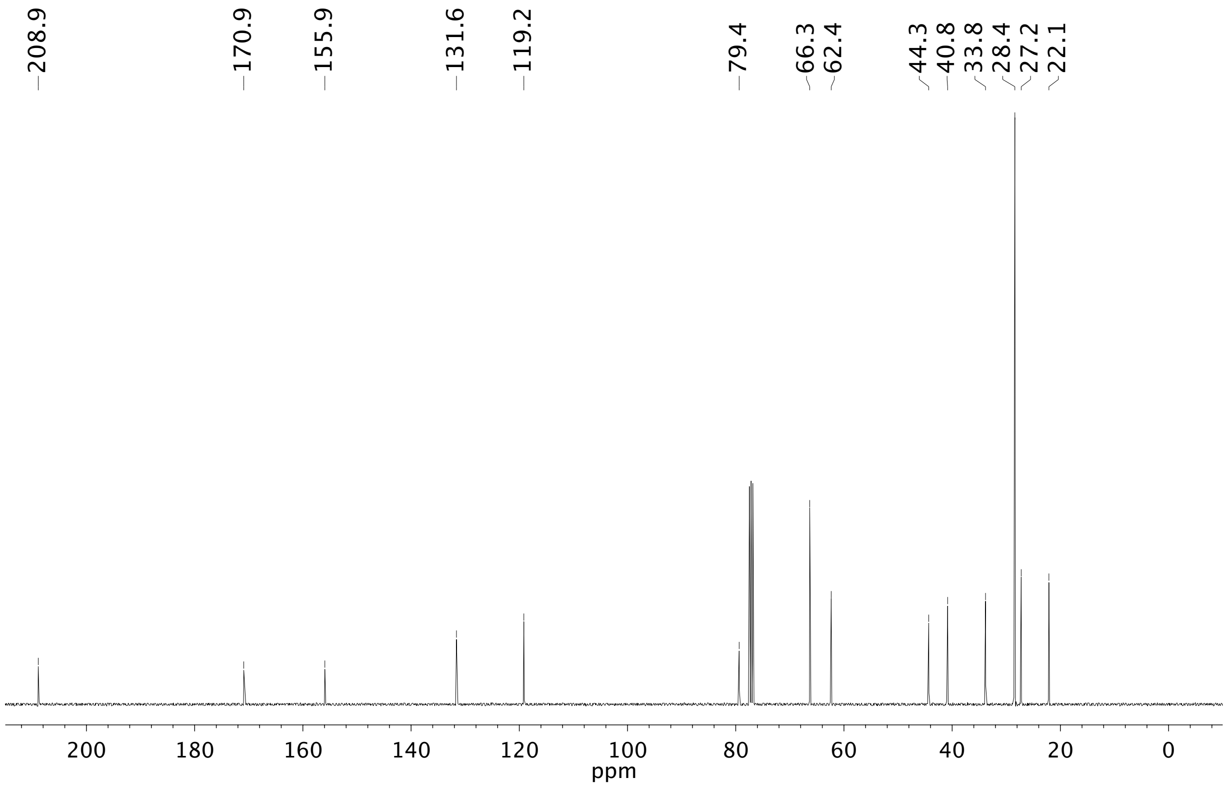


$^{13}C$ NMR (100 MHz, $CDCl_3$) of compound **11**.

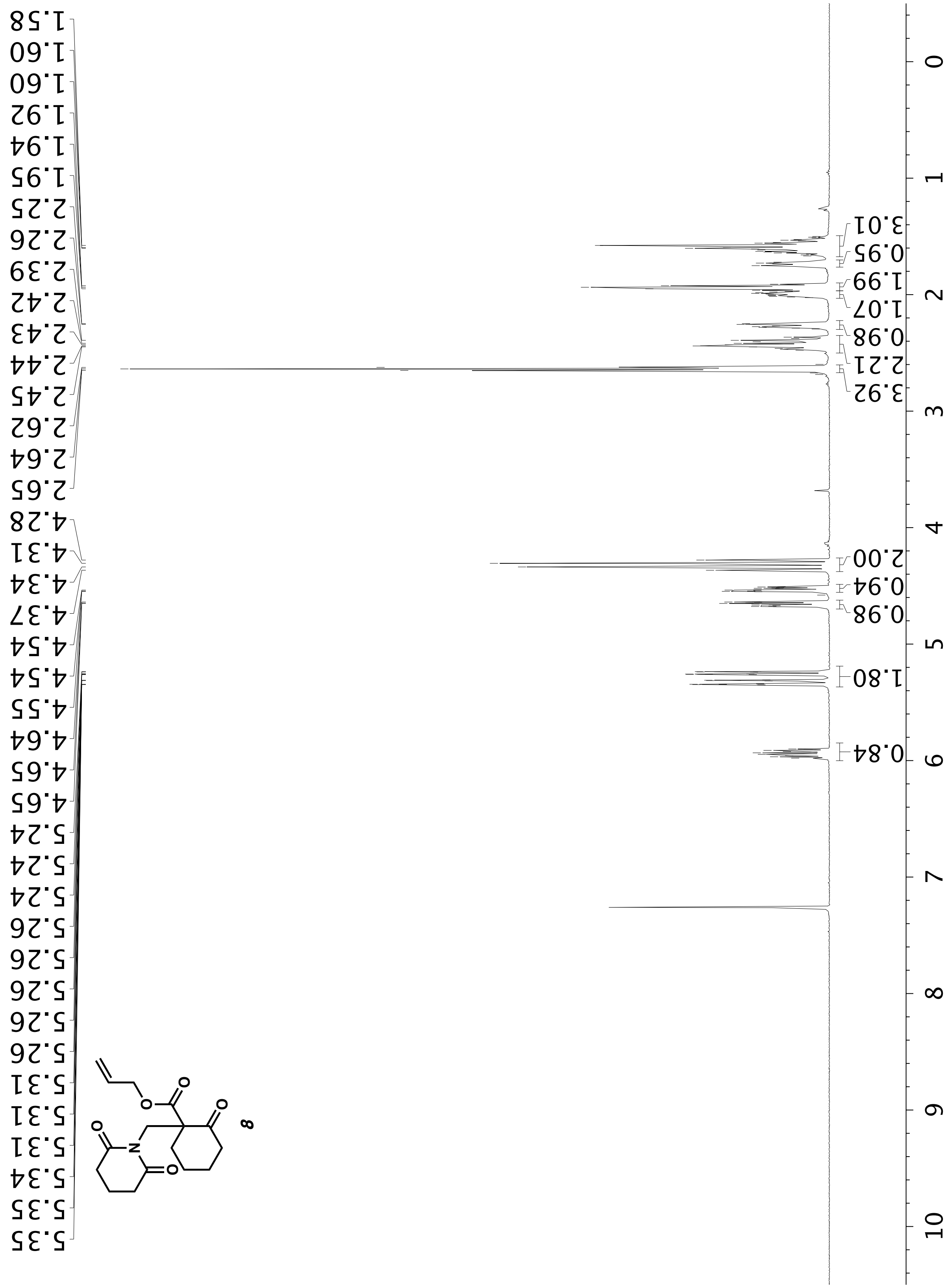

$^1H$ NMR (500 MHz, $CDCl_3$) of compound **8**.

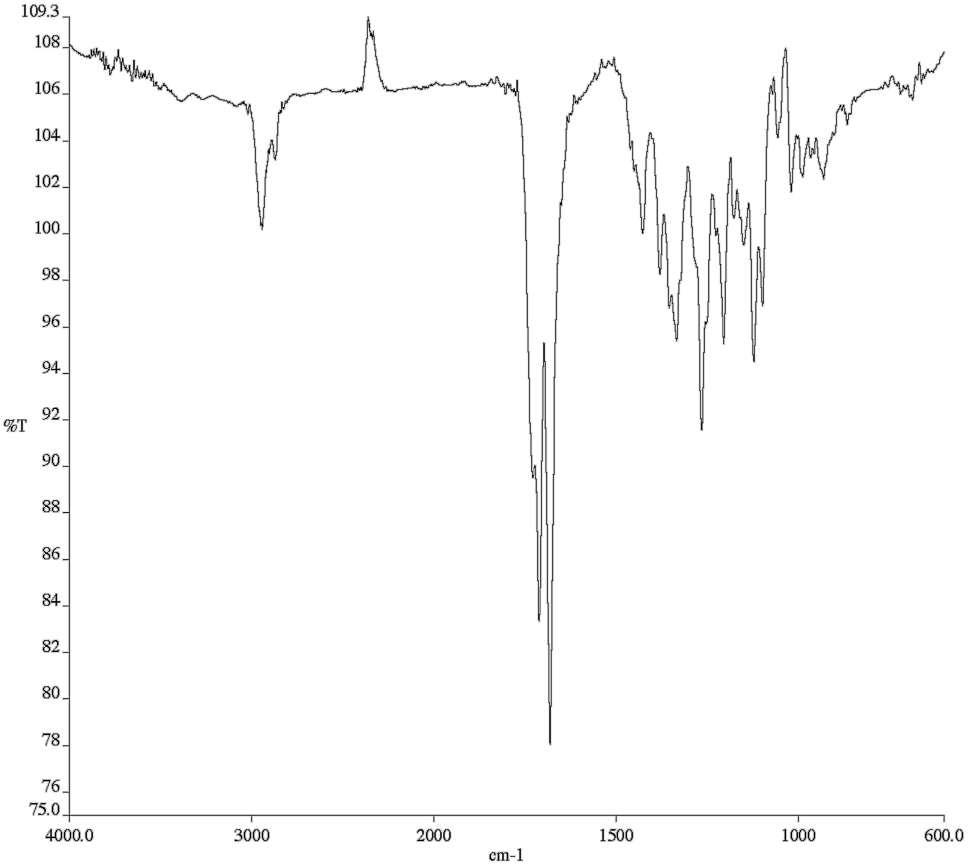


Infrared spectrum (Thin Film, NaCl) of compound **8**.

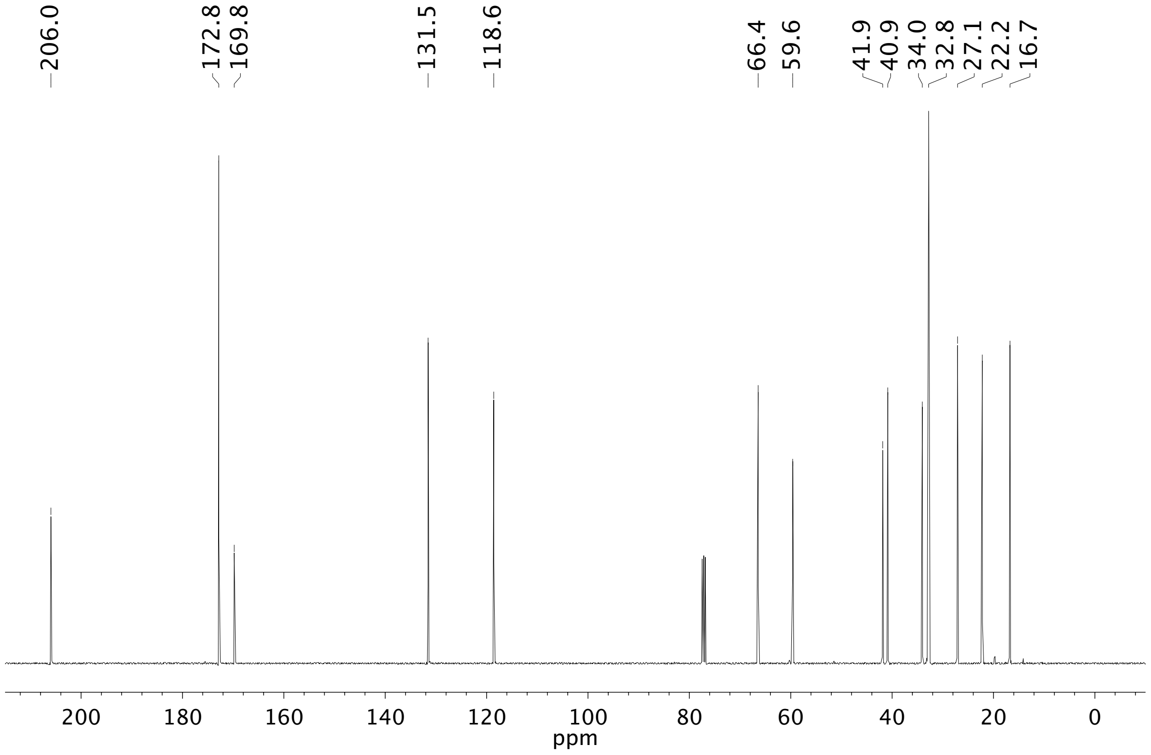


$^{13}C$ NMR (100 MHz, $CDCl_3$) of compound **8**.

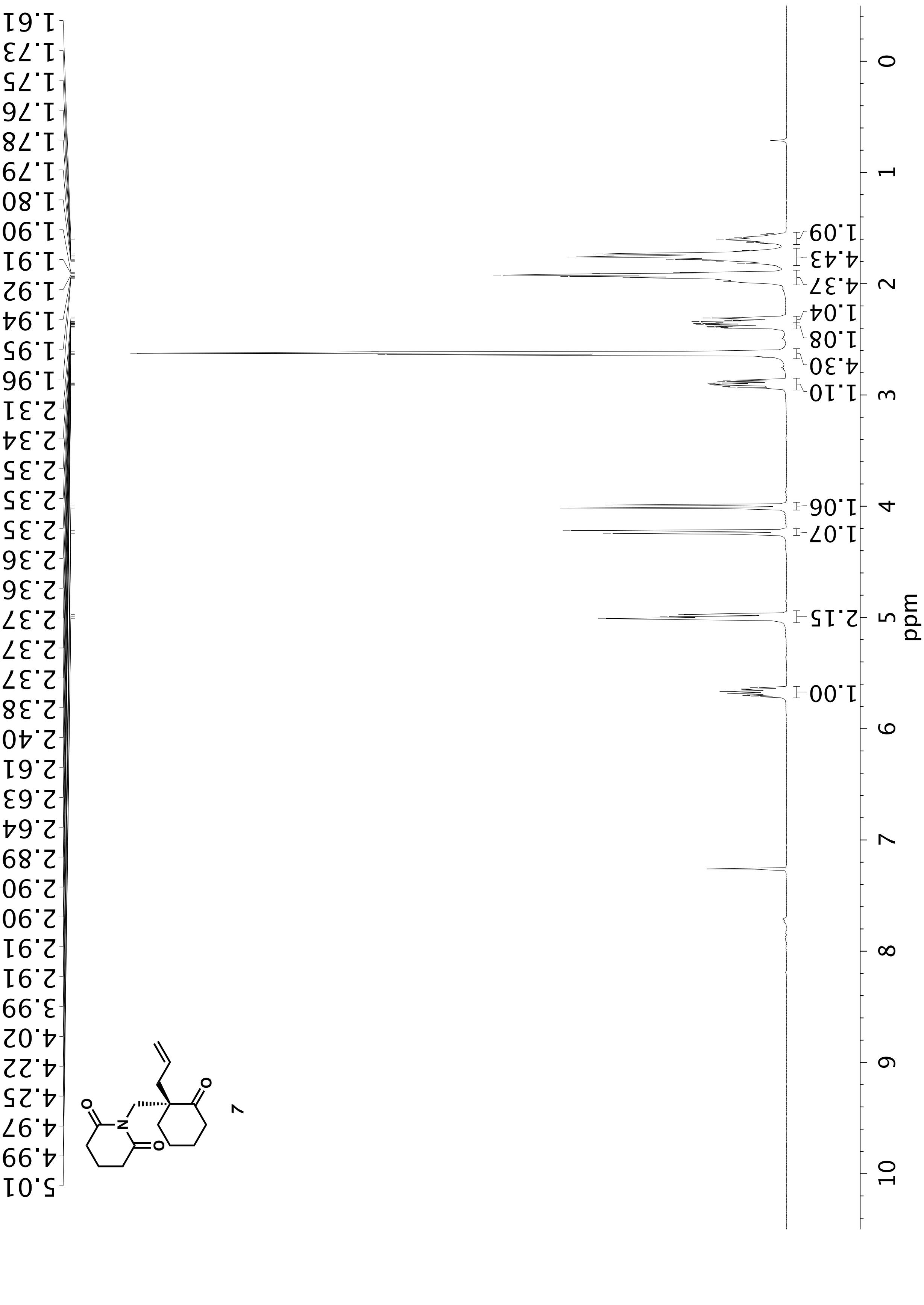


$^1$H NMR (500 MHz, $CDCl_3$) of compound **7.**

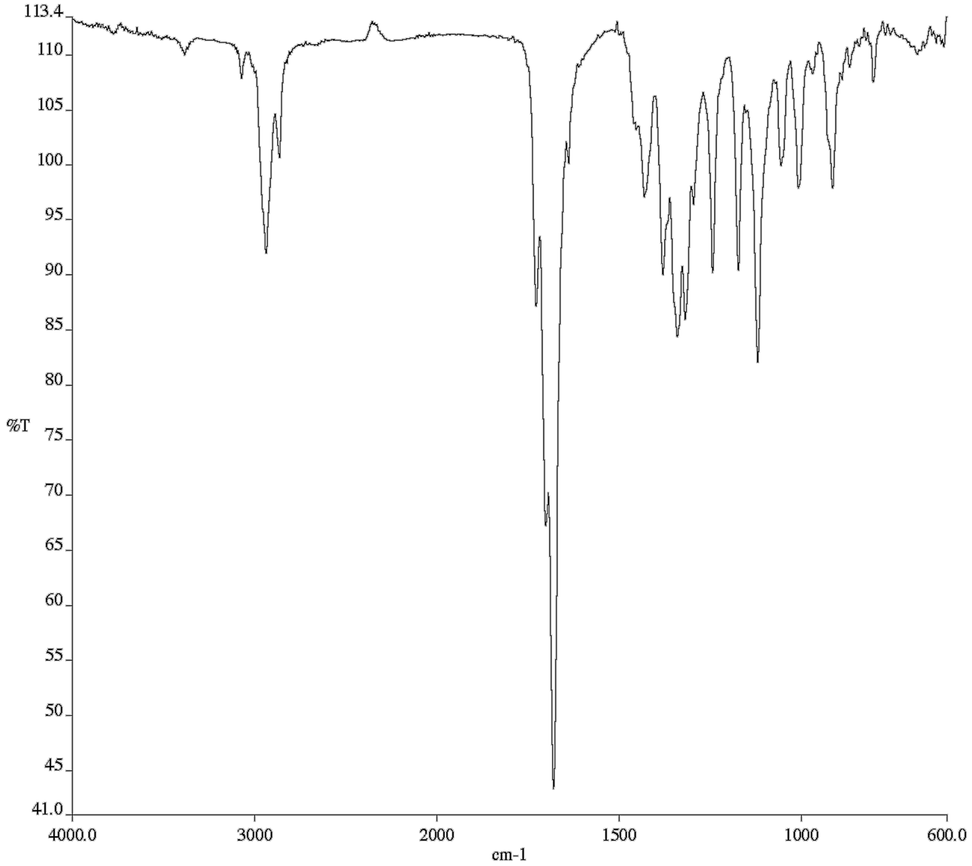


Infrared spectrum (Thin Film, NaCl) of compound **7**.

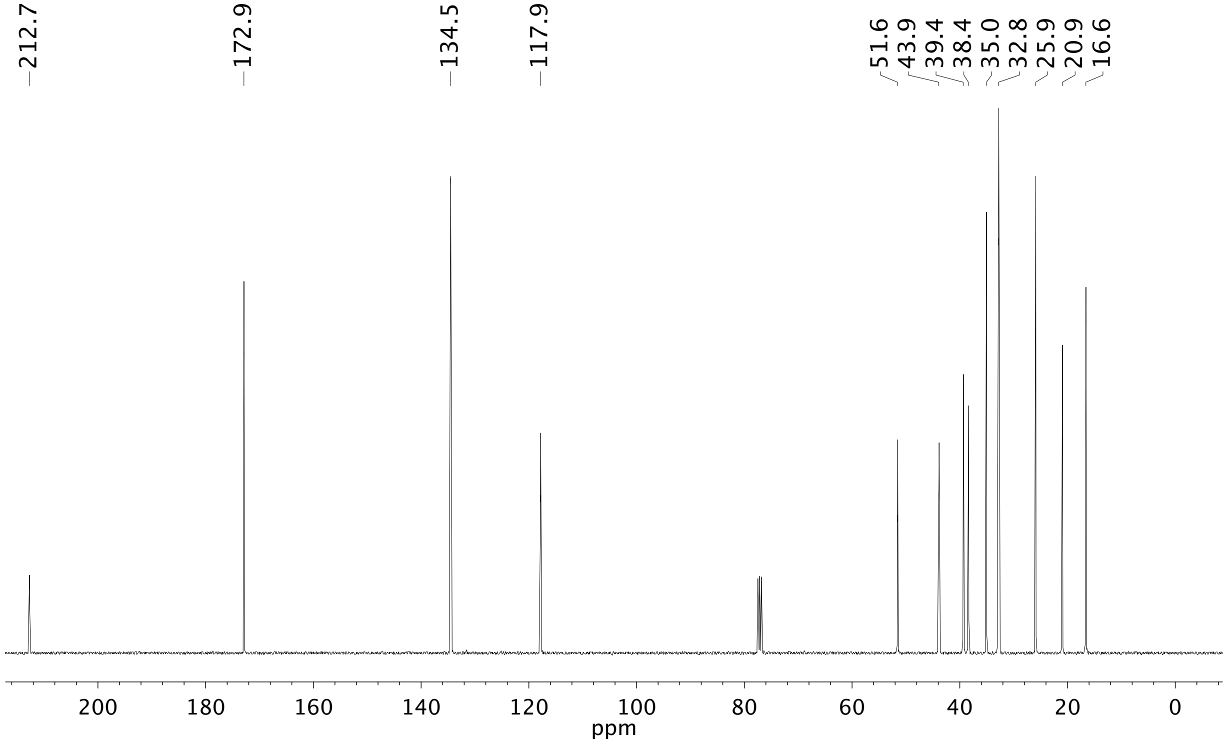


$^{13}C$ NMR (100 MHz, $CDCl_3$) of compound **7.**

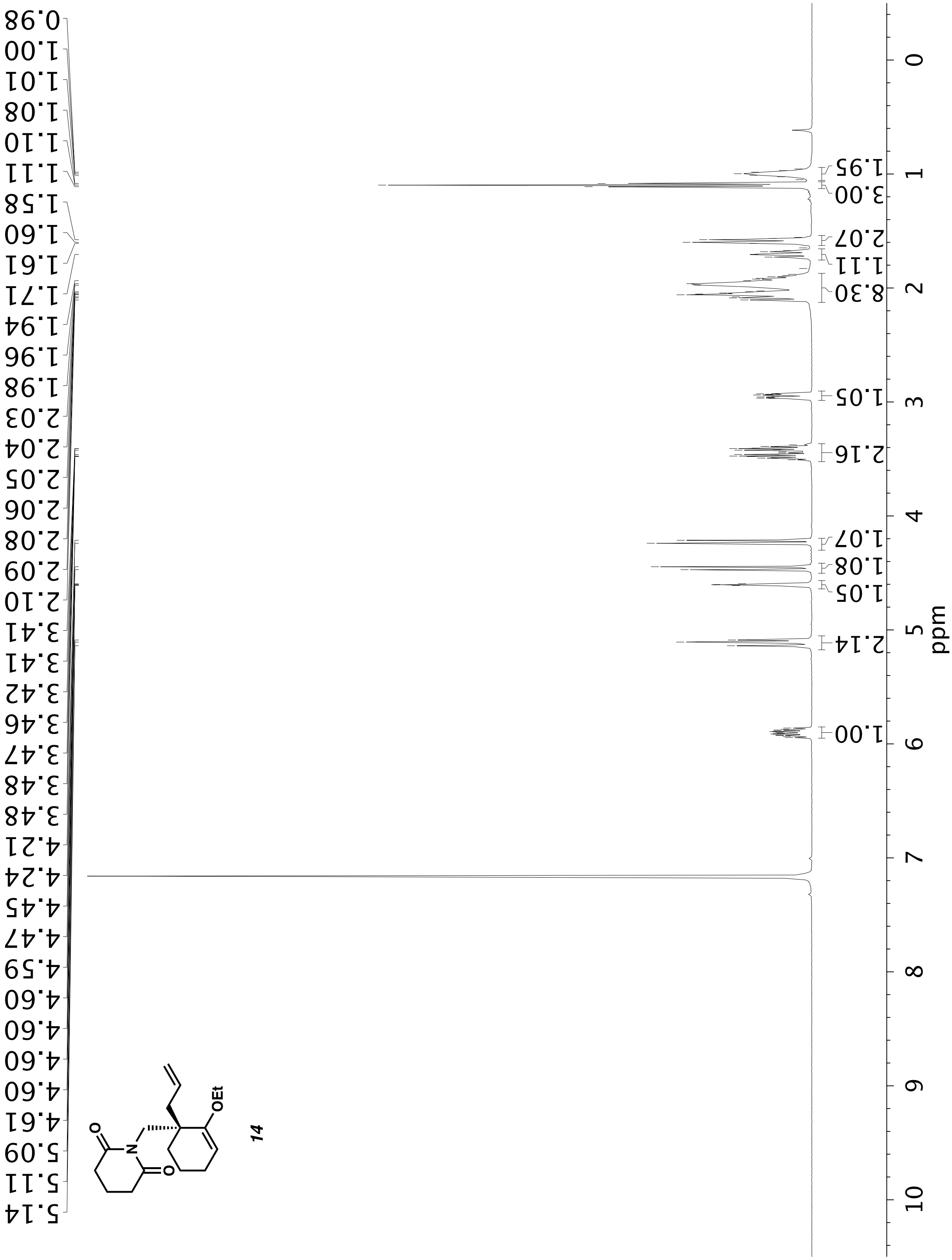


**14**

$^{1}$H NMR (500 MHz, $C_6D_6$) of compound **14.**

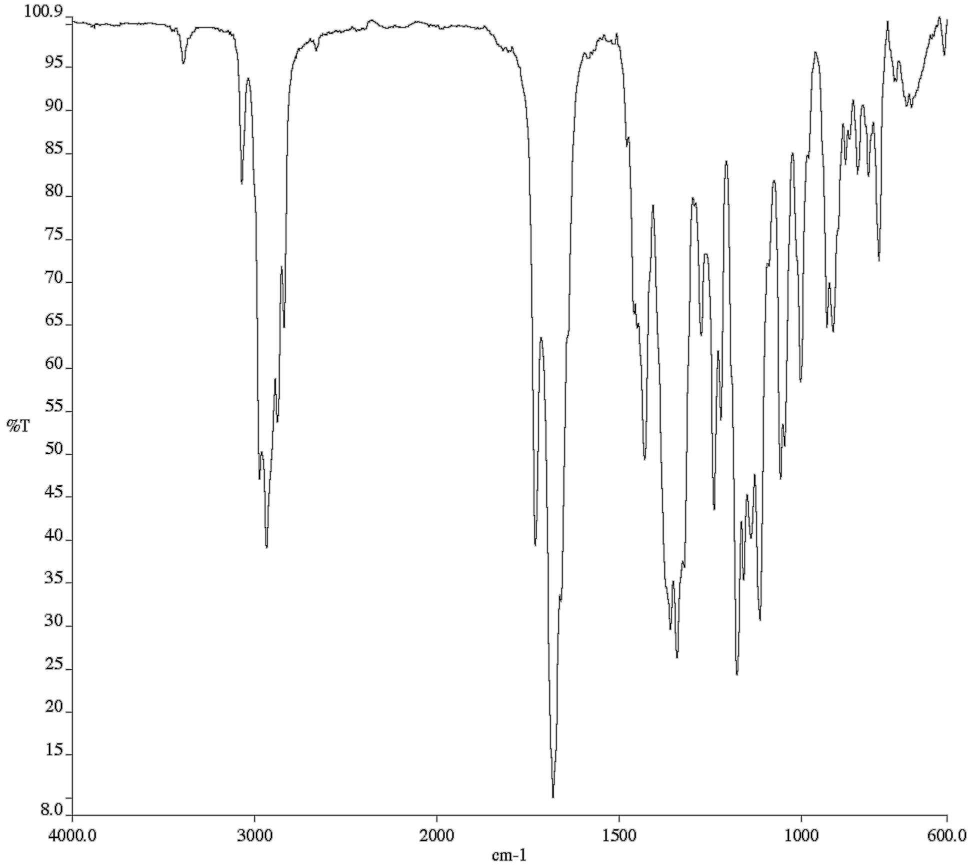


Infrared spectrum (Thin Film, NaCl) of compound **14**.

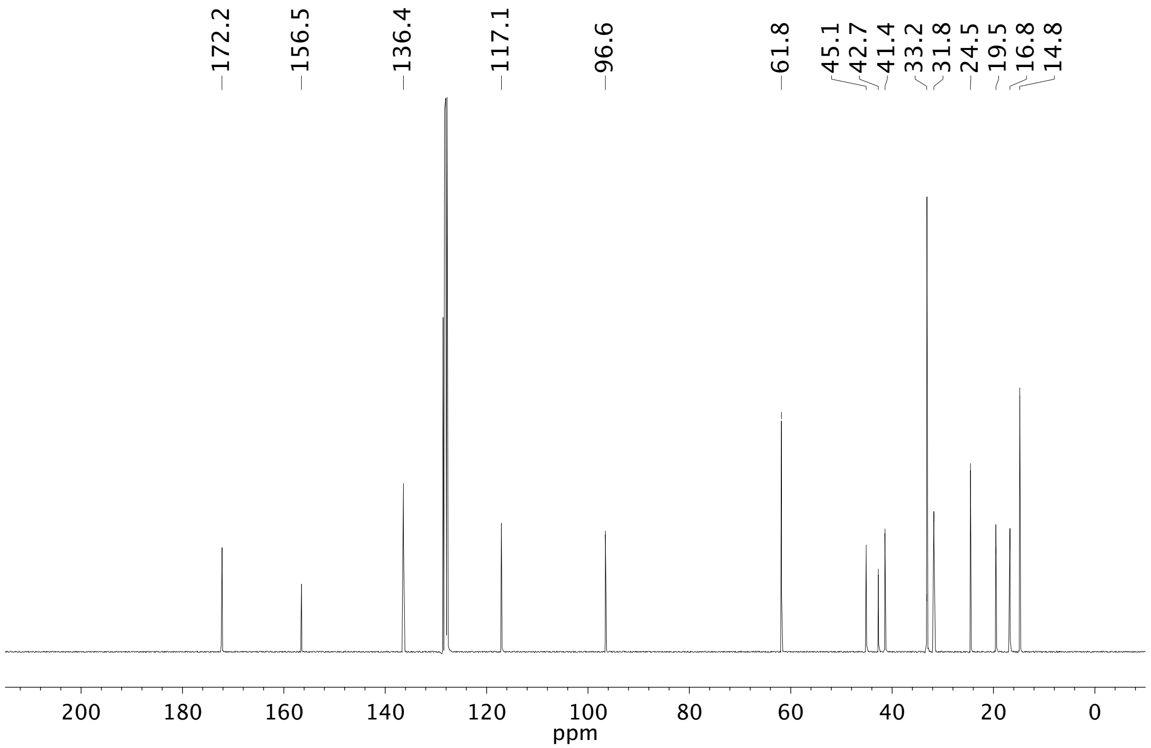


$^{13}C$ NMR (100 MHz, $C_6D_6$) of compound **14**.

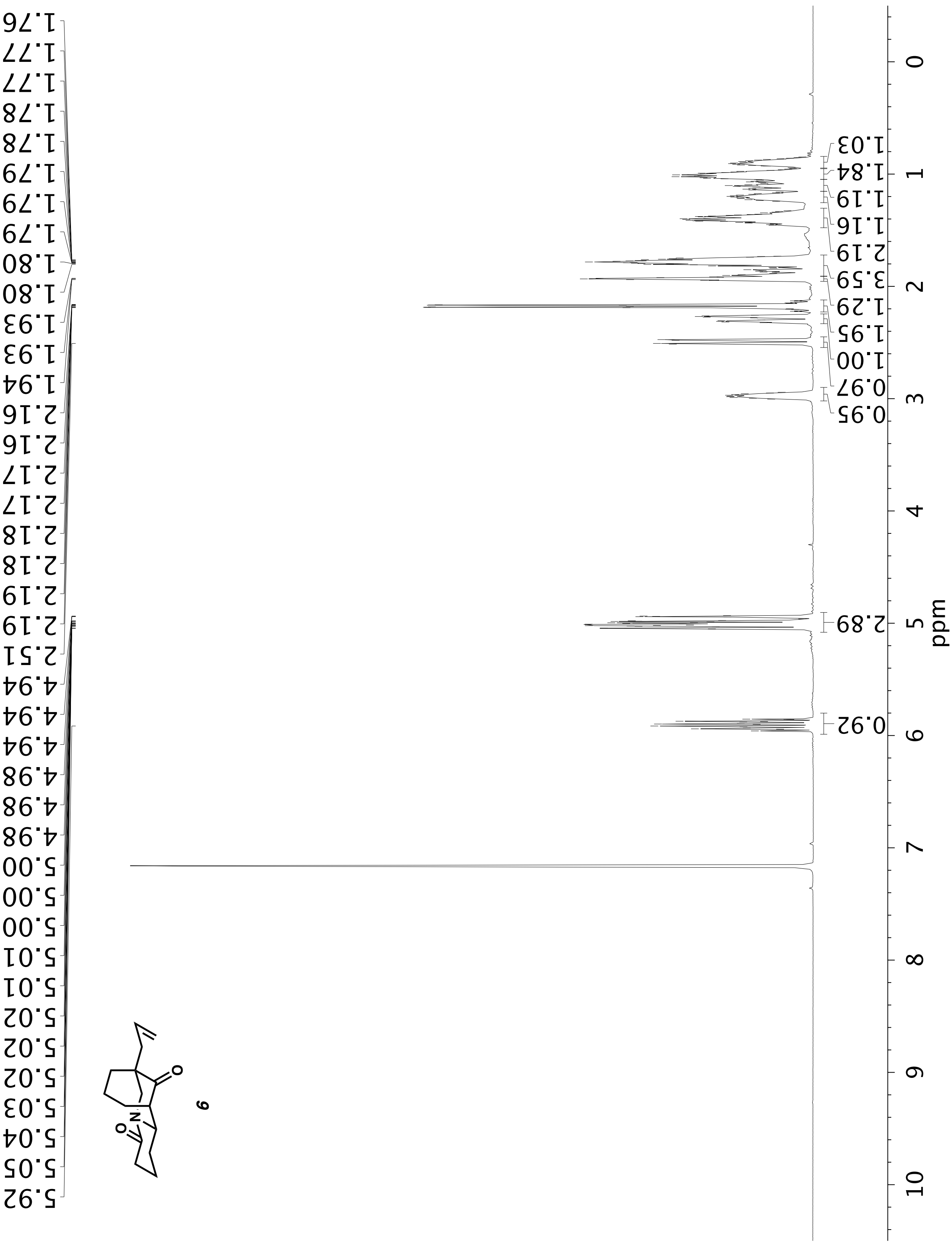


$^1$H NMR (500 MHz, $C_6D_6$) of compound **6.**

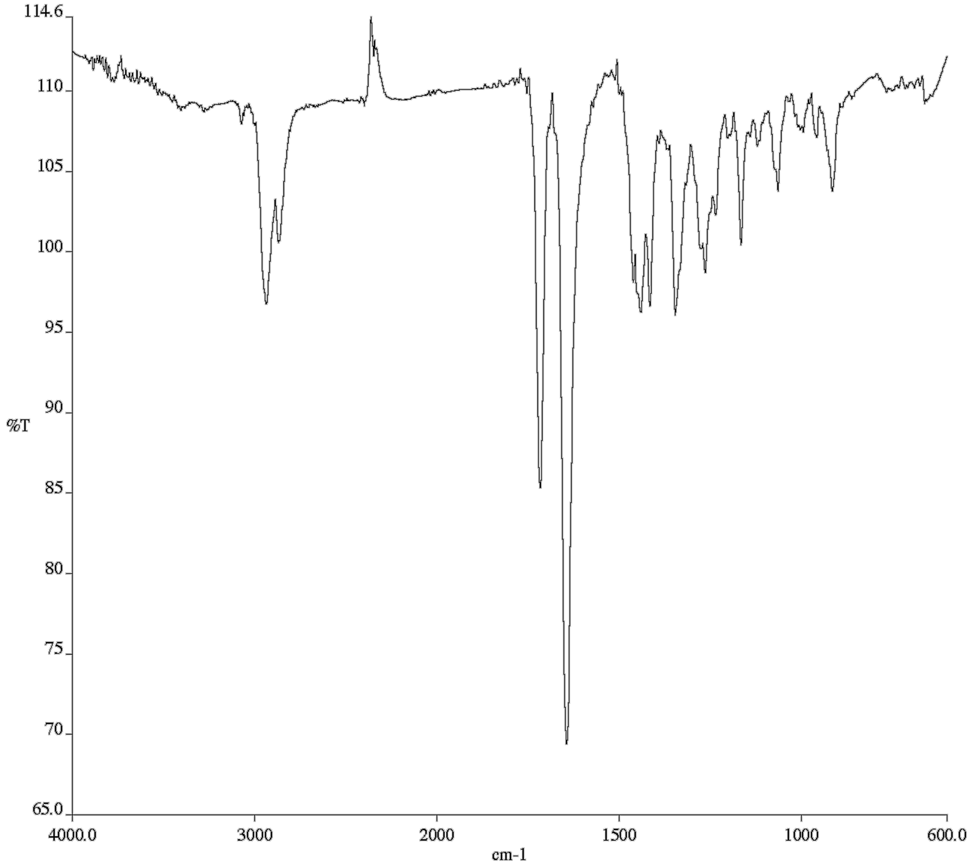


Infrared spectrum (Thin Film, NaCl) of compound **6**.

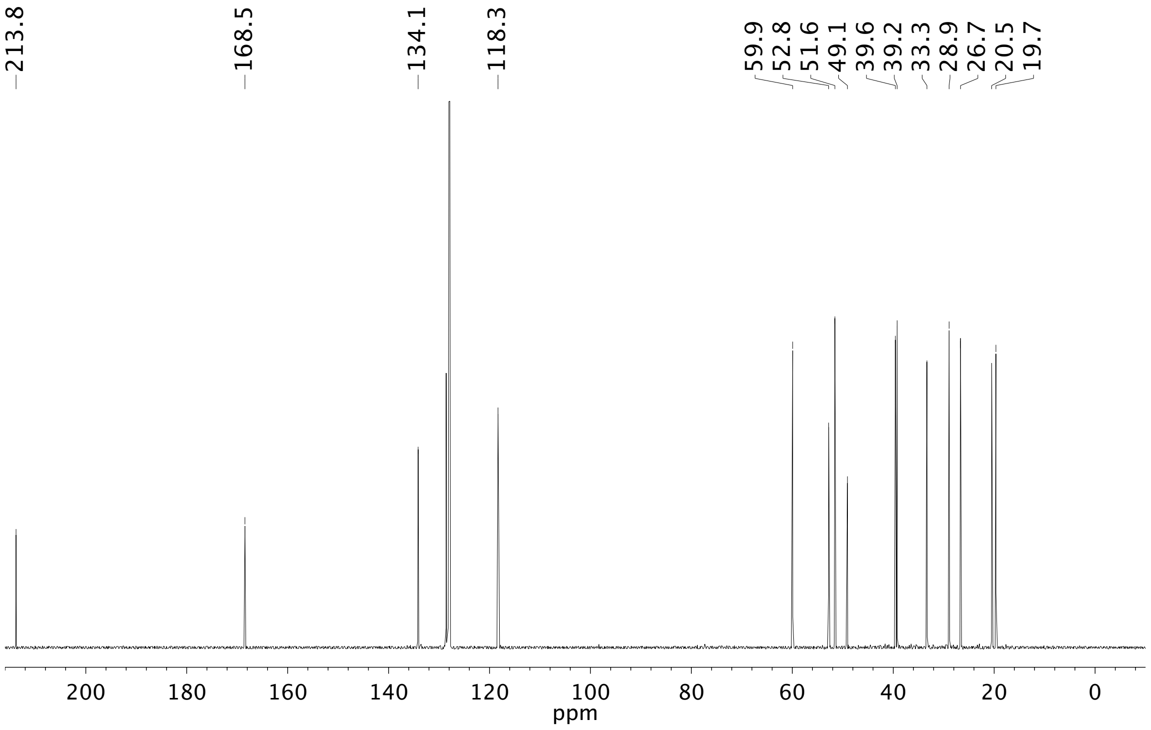


$^{13}C$ NMR (100 MHz, $CDCl_3$) of compound **6**.

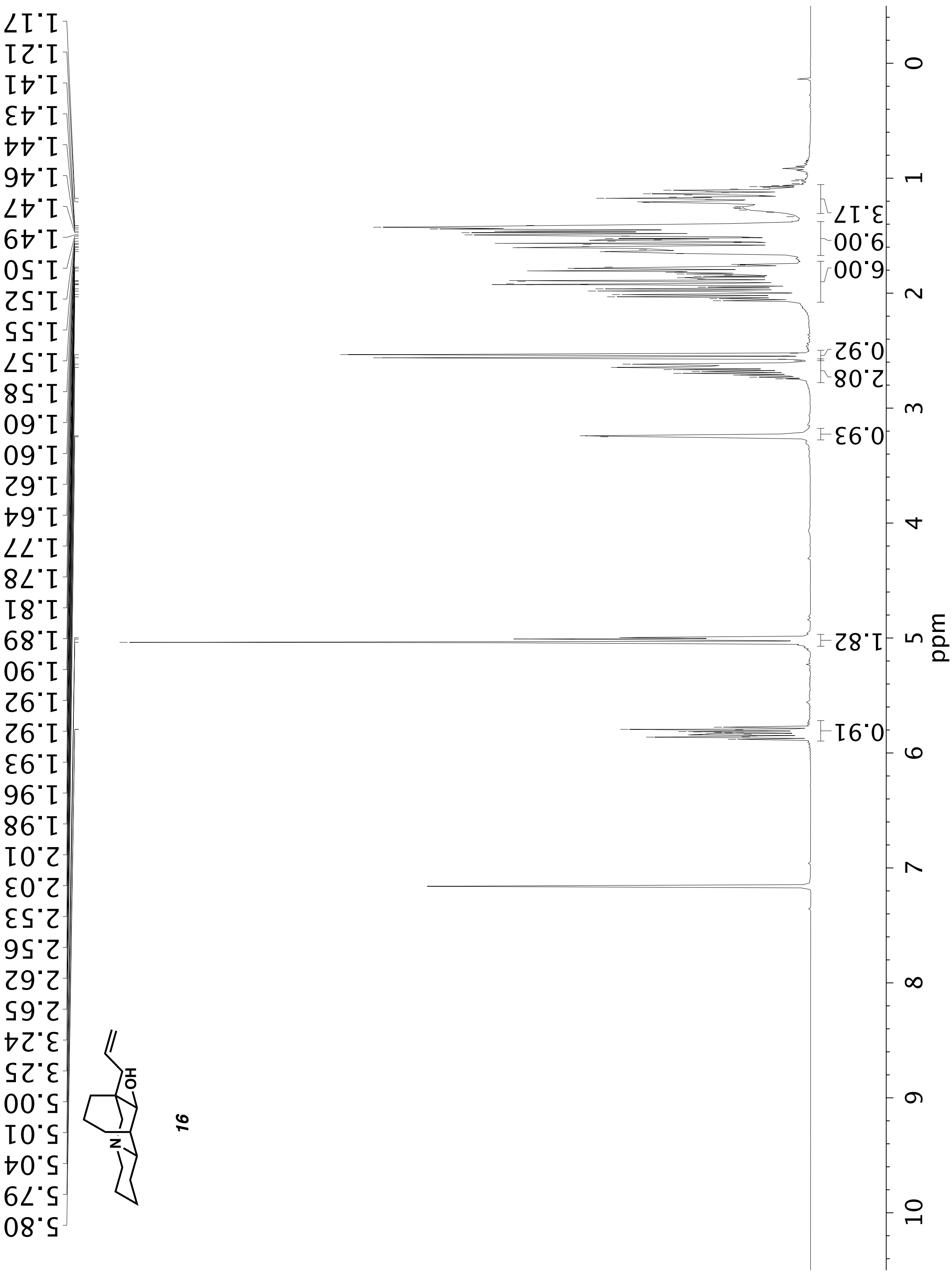


$^{1}H$ NMR (500 MHz, $C_6D_6$) of compound **16.**

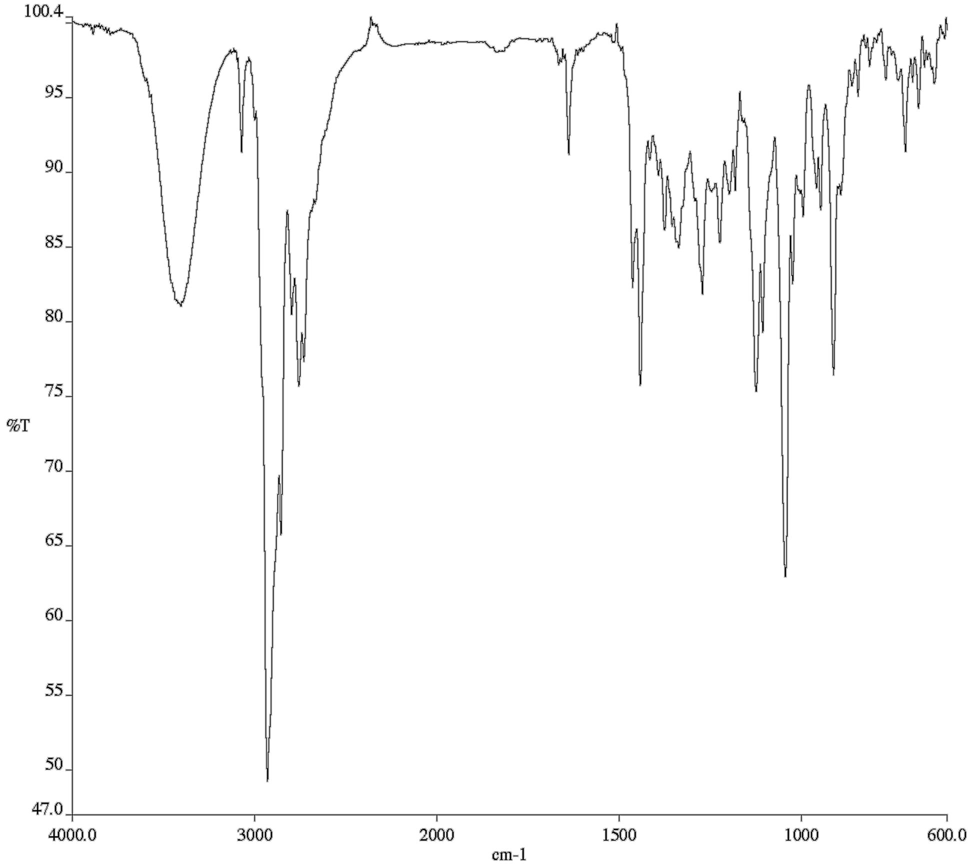


Infrared spectrum (Thin Film, NaCl) of compound **16**.

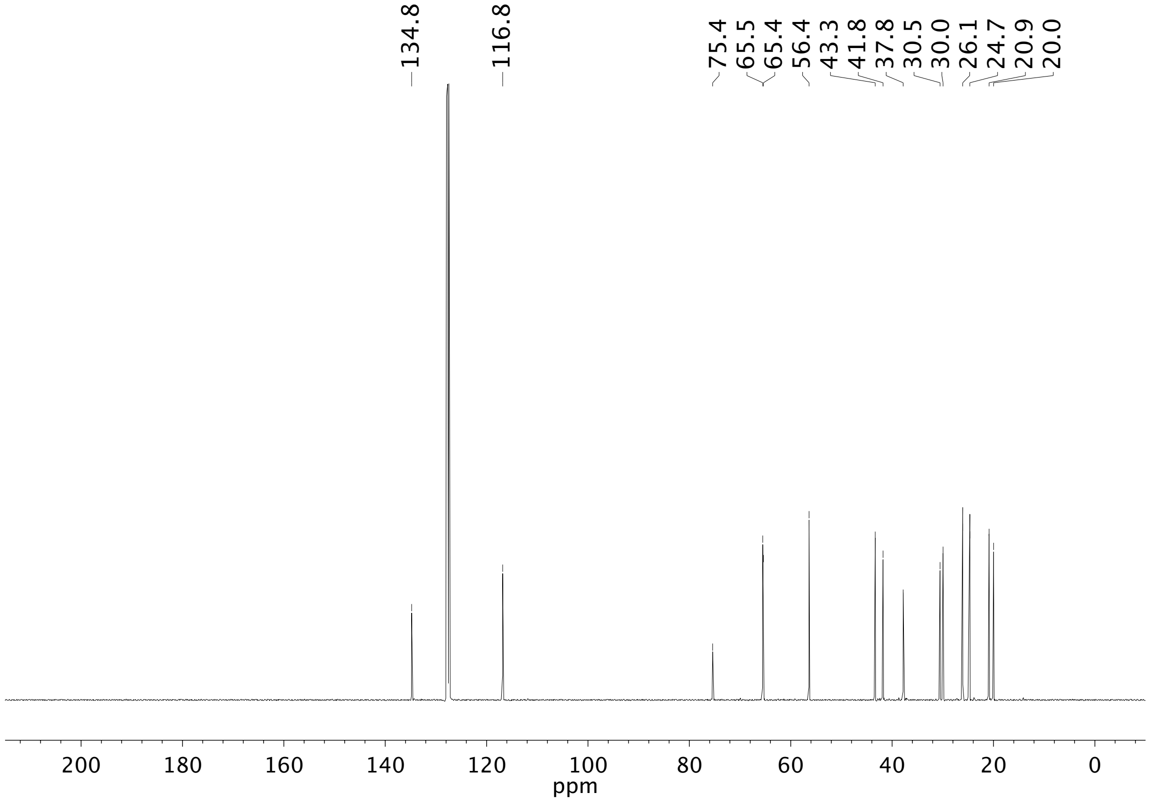


$^{13}C$ NMR (100 MHz, $CDCl_3$) of compound **16.**

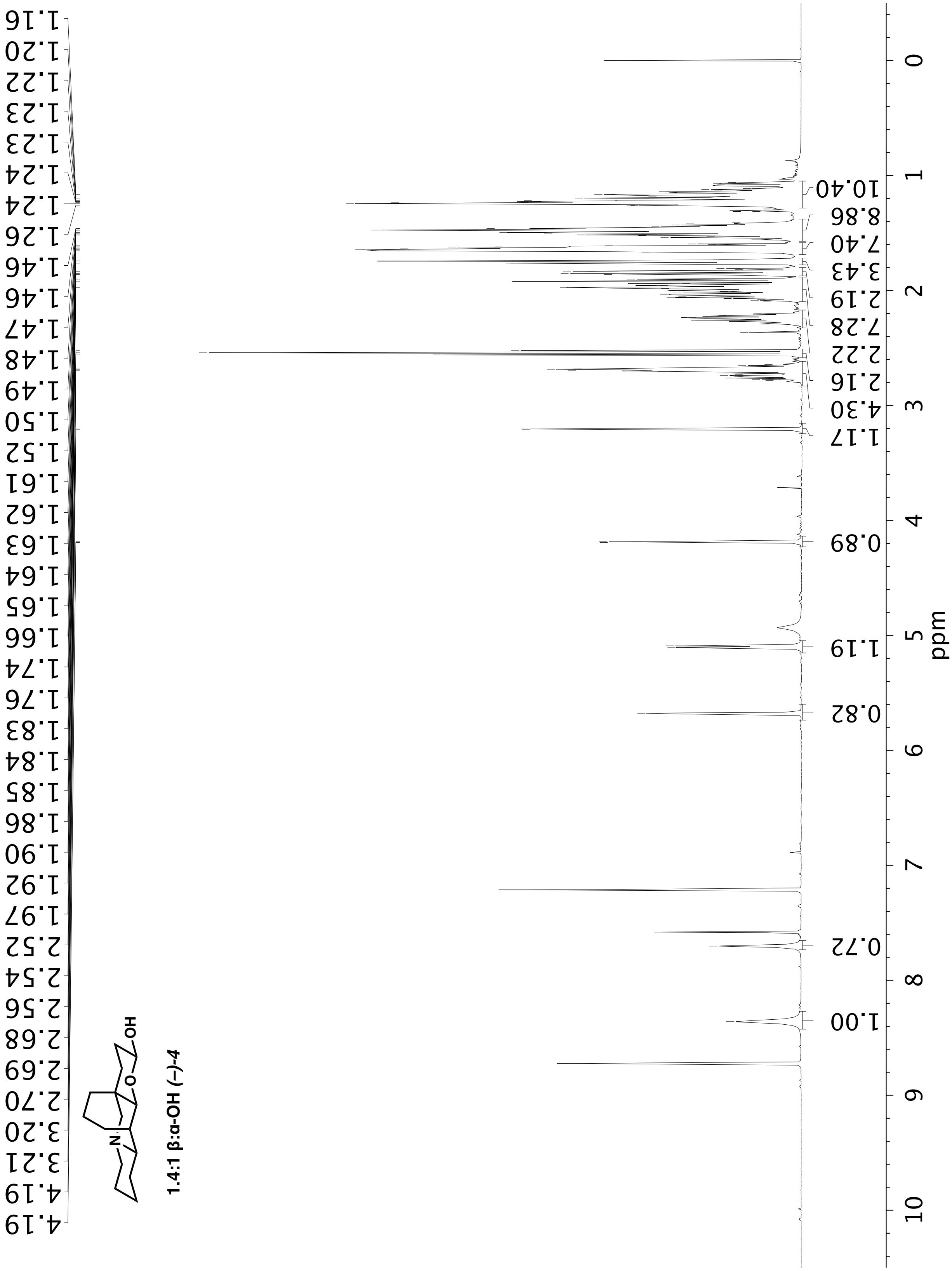


$^1$H NMR (600 MHz, pyridine-$d_5$) of compound **(–)-4**.

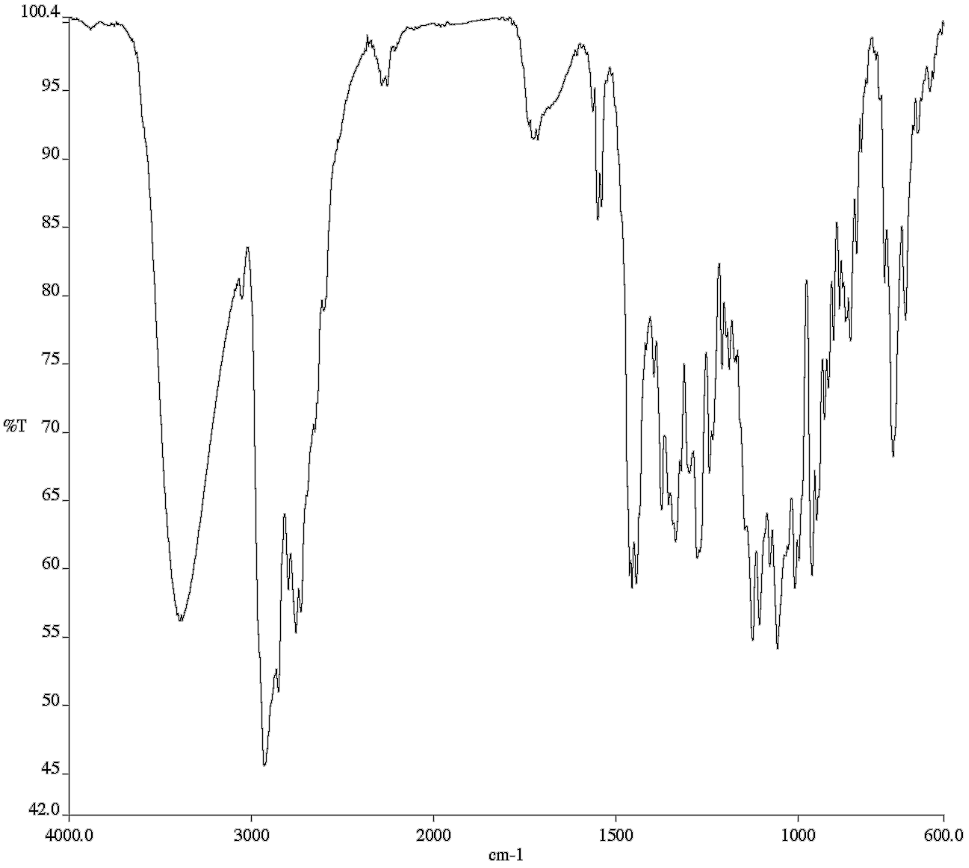


Infrared spectrum (Thin Film, NaCl) of compound **(–)-4**.

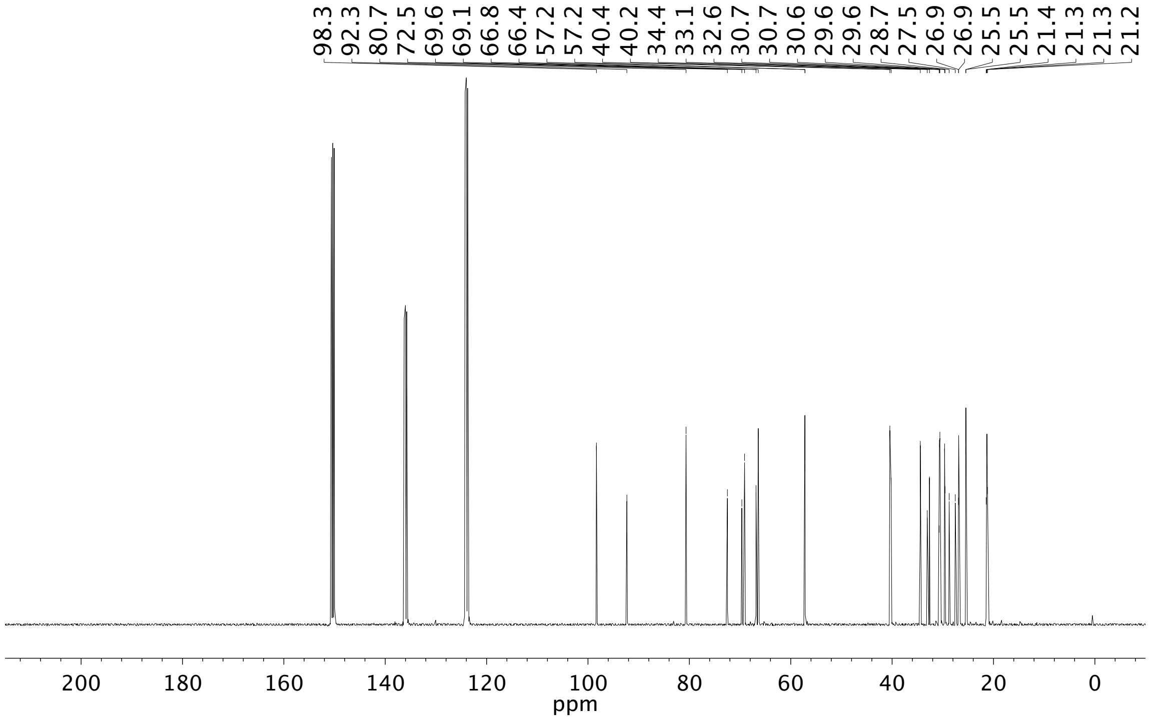


$^{13}$C NMR (100 MHz, pyridine-$d_5$) of compound **(–)-4**.

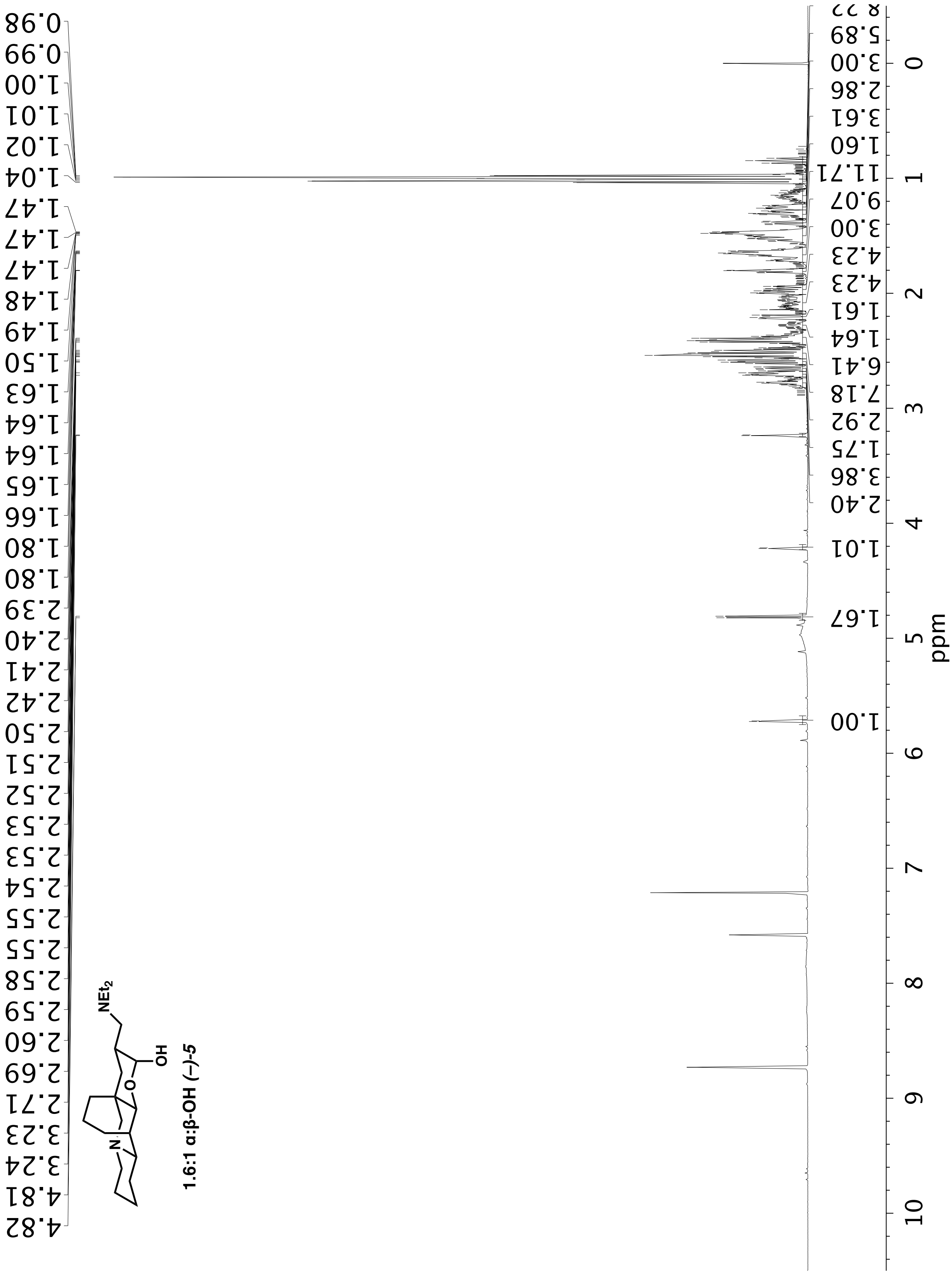


$^1$H NMR (600 MHz, pyridine-$d_5$) of compound **(–)-5.**

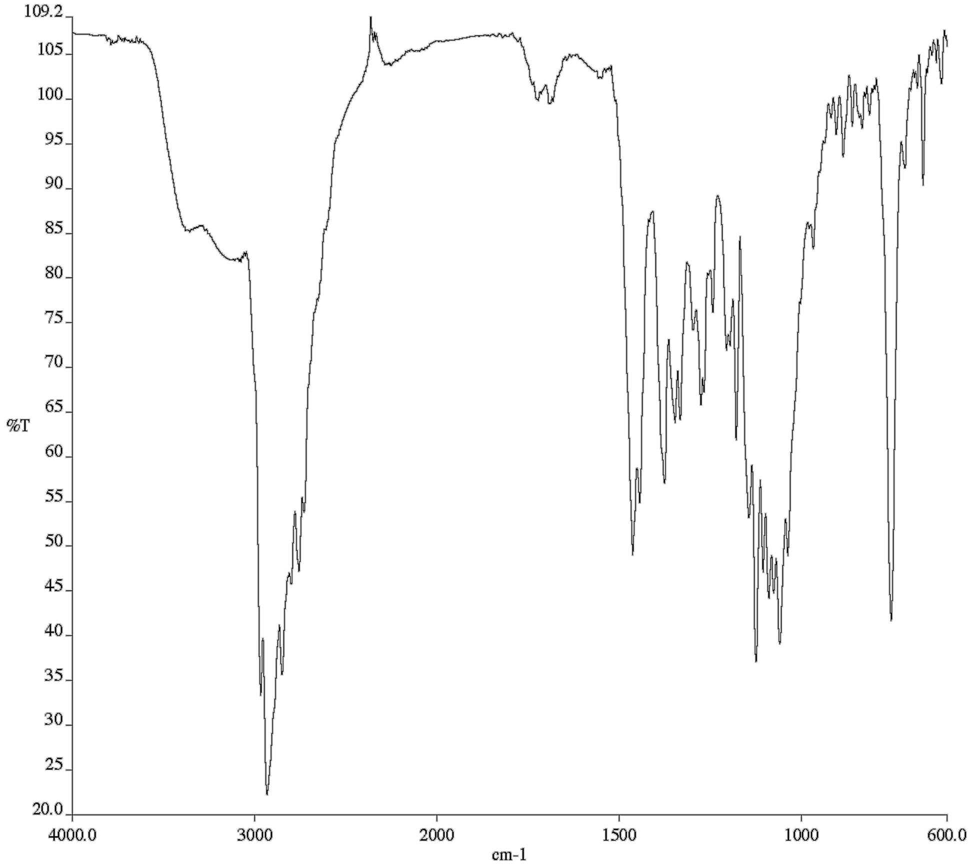


Infrared spectrum (Thin Film, NaCl) of compound **(–)-5**.

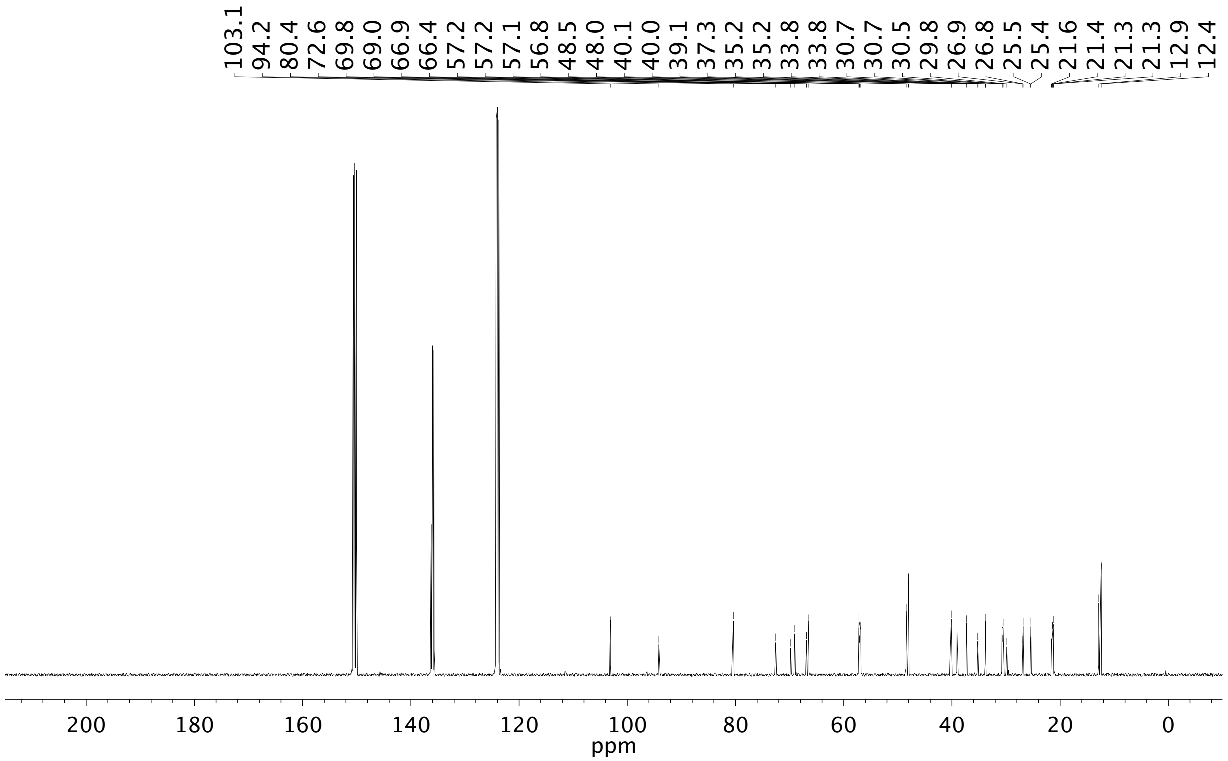


$^{13}C$ NMR (100 MHz, pyridine-$d_5$) of compound **(–)-5**.
